# Antimicrobial Peptides Increase Line Tension in Raft-Forming Lipid Membranes

**DOI:** 10.1021/jacs.4c05377

**Published:** 2024-07-17

**Authors:** Vladimir Rosenov Koynarev, Kari Kristine Almåsvold Borgos, Joachim Kohlbrecher, Lionel Porcar, Josefine Eilsø Nielsen, Reidar Lund

**Affiliations:** † Department of Chemistry, University of Oslo, Postboks 1033 Blindern, 0315 Oslo, Norway; ‡ Laboratory for Neutron Scattering and Imaging, Paul Scherrer Institut, Villigen 5232, Switzerland; ¶ Institut Laue-Langevin, 71 Av. des Martyrs, 38000 Grenoble, France; § Hylleraas Centre for Quantum Molecular Sciences, University of Oslo, Postboks 1033 Blindern, 0315 Oslo, Norway

## Abstract

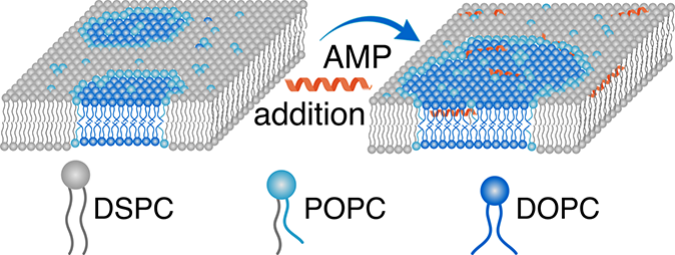

The formation of phase separated membrane domains is believed to be essential for the function of the cell. The precise composition and physical properties of lipid bilayer domains play crucial roles in regulating protein activity and governing cellular processes. Perturbation of the domain structure in human cells can be related to neurodegenerative diseases and cancer. Lipid rafts are also believed to be essential in bacteria, potentially serving as targets for antibiotics. An important question is how the membrane domain structure is affected by bioactive and therapeutic molecules, such as surface-active peptides, which target cellular membranes. Here we focus on antimicrobial peptides (AMPs), crucial components of the innate immune system, to gain insights into their interaction with model lipid membranes containing domains. Using small-angle neutron/X-ray scattering (SANS/SAXS), we show that the addition of several natural AMPs (indolicidin, LL-37, magainin II, and aurein 2.2) causes substantial growth and restructuring of the domains, which corresponds to increased line tension. Contrast variation SANS and SAXS results demonstrate that the peptide inserts evenly in both phases, and the increased line tension can be related to preferential and concentration dependent thinning of the unsaturated membrane phase. We speculate that the lateral restructuring caused by the AMPs may have important consequences in affecting physiological functions of real cells. This work thus shines important light onto the complex interactions and lateral (re)organization in lipid membranes, which is relevant for a molecular understanding of diseases and the action of antibiotics.

## Introduction

Lateral phase separation of the cellular membrane into small and dynamic lipid domains plays a key role in the physiological function of the cell. These domains are often termed rafts and differ in lipid composition from the surrounding continuous phase. Although the existence of such rafts in living cells has been questioned^1,2^ and critically debated,^3^ they are now more widely accepted and have been linked to a variety of different cellular processes. Almost since the inception of the term lipid raft by Simons and Ikonen in 1997,^4^ the prevailing theory has been that signal transduction proteins in eukaryotic cell membranes are arranged into membrane rafts enriched in particular lipids such as cholesterol and sphingolipids.^5^ More recent work has related lipid rafts to membrane protein conformation,^6^ immune signaling,^7^ viral interactions,^8^ cardiovascular decease,^9,10^ and cancer.^11^ Until relatively recently, the formation of lipid rafts was believed to be a fundamental step in the evolution of more complex cells, and more primitive cells such as bacteria and archaea therefore do not exhibit sophisticated organization of their cellular membranes. However, it has now been shown that bacterial processes associated with transport, protein secretion, and signal transduction often occur in functional membrane domains similar to rafts.^12−14^ The importance of understanding the formation and structure of lipid rafts is demonstrated by the broad range of physiological and pathological processes with which they are hypothesized to be associated.

Some of the key questions are, therefore, how the raft structure is affected by changes in the environment or by exposure to bioactive and therapeutic molecules. A particularly interesting and relevant question is how surface-active peptides, which are known to interact with and even perturb the cellular membrane, affect the lateral lipid organization. This large and rather diverse group of molecules includes both amyloid-forming peptides, linked to human neurological diseases such as Alzheimer’s and Parkinson’s disease,^15^ and antimicrobial peptides (AMPs). AMPs are short and largely cationic polypeptides that tend to have broad spectrum activity against several different classes of pathogens, including bacteria, viruses, and fungi.^16,17^ They are often found to affect the lipid packing and cause membrane thinning/thickening or even detergent-like solubilization of the membrane. More subtle effects, such as increased lipid flip-flop, have also been attributed to AMPs.^18^ Similarly, amyloid-forming peptides have been shown to induce membrane thinning and solubilization, as well as membrane remodeling.^19^

Given the ability of many peptides to interact with the cellular membrane and cause structural changes, it is likely that they also affect lipid rafts and the lateral organization of the membrane. There is emerging evidence that lipid rafts play a role in the processing of amyloid-β (Aβ) peptide^20^ and that the presence of lipid domains might enhance Aβ–membrane interactions.^21^ There is also some evidence that the presence of lipid rafts affects the selectivity and efficacy of AMPs.^22,23^ Although these initial studies provide some insight, the interactions between lipid rafts and surface-active peptides, particularly AMPs, remain poorly understood.

Much of the current understanding of membrane phase separation and lipid rafts stems from studies of model lipid membranes.^6,24^ Although the importance of studies in live cells cannot be overstated, the ability to control the precise structure and composition of model membranes allows for a more detailed understanding of the subtle biophysical and thermodynamic processes governing the rafts. It is largely held that lipid rafts form due to the immiscibility of the different lipids comprising the membrane. This can be traced back to a difference in the length and extension of the acyl chains of saturated and unsaturated lipids, particularly in the presence of cholesterol. The energetic cost of mixing lipids of different lengths drives phase separation and gives rise to line tension between two phases of different thicknesses.^25−30^

Rafts are readily observed in membranes containing both saturated and unsaturated lipids as well as cholesterol. The saturated lipids and cholesterol form a liquid ordered (*L*_o_) phase, while the unsaturated lipids form a liquid disordered (*L*_d_) phase. The specific phase behavior of such ternary lipid mixtures has been extensively explored by using giant unilamellar vesicles (GUVs) and fluorescence microscopy. Veatch and Keller investigated many different combinations of saturated and unsaturated phosphocoline (PC) lipids and cholesterol in 2003^25^ and with sphingomyelin (SM) instead of saturated PC in 2005.^26^ They demonstrated the formation of highly dynamic domains with varying morphologies, and they point to line tension being the driving factor for phase separation. They further report a linear relationship between the miscibility transition temperatures and melting temperature of the saturated lipid, which is directly linked to the acyl chain length. These initial studies suggested that diunsaturated DOPC (1,2-dioleoyl-*sn*-glycero-3-phosphocholine) readily forms microsized rafts in mixtures with saturated PC and cholesterol over a wide range of temperatures and compositions. On the other hand, using the monounsaturated POPC (1-palmitoyl-2-oleoyl-*sn*-glycero-3-phosphocholine) instead of DOPC seemingly did not result in domains.

Following this work, Heberle et al.^28^ used fluorescence resonance energy transfer (FRET) to show that lipid membranes composed of POPC in addition to DSPC (1,2-distearoyl-*sn*-glycero-3-phosphocholine) and cholesterol also form lipid domains, but in the nanometer size range and below the optical resolution limit. They report a similar phase diagram to the DSPC/DOPC/cholesterol diagram previously established,^27^ but with the notable difference in domain size.

In addition to surface sensitive methods, such as AFM used in combination with supported bilayers,^30^ optical methods, such as fluorescence microscopy, have been instrumental in establishing detailed phase diagrams of these systems. However, the inability of optical techniques to resolve nanosized domains poses a significant limitation, especially considering that cellular rafts are likely to be in the nanometer range.^6,31^ To this end, small-angle X-ray and neutron scattering (SAXS and SANS) methods are of great utility due to their ability to resolve structures between a few ångströms and several hundred nanometers. SANS in particular can be used to visualize and characterize nanosized domains in freely floating vesicles, as demonstrated in several recent works.^29,32,33^ Based on the detailed phase diagram first presented by Konyakhina et al.,^34^ Heberle et al.^29^ used SANS and selective isotope labeling to investigate the structure of lipid rafts in small unilamellar vesicles (SUVs) with a quaternary lipid composition of DSPC, POPC, DOPC, and cholesterol. They report the formation of nanoscale rafts and demonstrate how the domain size can be controlled by varying the fraction of DOPC, with higher amounts of DOPC resulting in larger rafts. Furthermore, they show that there is a significant difference in thickness between the two phases and that this difference also increases with increasing the DOPC fraction. This is attributed to the less extended acyl chains of the diunsaturated DOPC as compared to those of the monounsaturated POPC. The authors suggest that the difference in thickness is the main contributing factor to the observed line tension and resulting phase separation. The model system was further investigated by Nickels et al.^32^ using neutron spin echo (NSE), where the authors showed that the lipid rafts are registered across the two bilayer leaflets. Similar line tension driven phase separation was reported by García-Sáez et al.^30^ for supported lipid bilayers, where the thickness mismatch was varied using unsaturated PC lipids with varying acyl chain lengths.

Here we address the effect of antimicrobial peptides on the lipid organization and raft formation in model membranes using SANS and SAXS. Based on the seminal work by Heberle et al.,^29^ we systematically investigate a series of vesicles with rafts of various nanosizes and expose them to several different natural antimicrobial peptides. We chose AMPs that vary in structure, charge, length, and origin. These include indolicidin (ILPWKWPWWPWRR-NH2), an unstructured peptide originating from bovine neutrophils, the human α-helical cathelicidin LL-37, (LLGDFFRKSKEKIGKEFKRIVQRIKDFLRNLVPRTES), aurein 2.2 (GLFDIVKKVVGALGSL), and magainin II (GIGKFLHSAKKFGKAFVGEIMNS), which are both α-helical peptides that originate from the Australian bell frog (*Litoria aurea*) and the African clawed frog (*Xenopus laevis*), respectively. These peptides all exhibit broad spectrum antimicrobial activity, cytotoxicity at elevated concentrations, and their interactions with model membranes are well characterized.^18^ Using SAXS, we have previously showed that while indolicidin and magainin II insert into the outer leaflet of model membranes composed of saturated PC, phosphatidylglycerol (PG), and phosphoethanolamine (PE) lipids, aurein 2.2 and LL-37 rather insert in a transmembrane fashion depending on the peptide to lipid ratio.^18,35,36^ Independent of the positioning in the membrane, none of the peptides affected the thickness of the saturated lipid membranes; however, at higher ratios, some solubilization of the membrane was observed, especially in the case of LL-37^18^ or in membrane systems with higher contents of PE lipids.^37^ Interestingly, the results we present in this work show that, irrespective of the specific peptide sequence, the addition of AMPs leads to a significant growth of lipid rafts. We attribute the growth to increased line tension caused by preferential thinning of the unsaturated/disordered phase, while the thickness of the ordered phase is unaltered as the peptide inserts into the membrane. We hypothesize that the lateral restructuring observed in these model systems may have relevance to real cell membranes with consequences for physiological functions.

## Materials and Methods

### Sample Preparation

Large unilamellar vesicles (LUVs) with different well-defined lipid compositions were prepared and used in the presented SANS and SAXS studies. The LUVs were prepared using synthetic and high purity lipid powders from Avanti Polar Lipids, including DSPC (1,2-distearoyl-*sn*-glycero-3-phosphocholine), DOPC (1,2-dioleoyl-*sn*-glycero-3-phosphocholine), POPC (1-palmitoyl-2-oleoyl-*sn*-glycero-3-phosphocholine), DMPE-PEG (1,2-dimyristoyl-*sn*-glycero-3-phosphoethanolamine-*N*-[methoxy(polyethylene glycol)-2000]), and tail-deuterated DSPC-d70 (1,2-distearoyl-d70-*sn*-glycero-3-phosphocholine). Cholesterol was purchased from Sigma-Aldrich.

Based on the method and lipid compositions presented by Heberle et al.,^29^ three raft-forming lipid compositions were chosen to form LUVs with small, medium, or large rafts, respectively. These vesicles have a quaternary lipid mixture composed of saturated DSPC in equimolar ratio to the monounsaturated POPC and diunsaturated DOPC combined, as well as a fixed molar fraction of cholesterol. An advantage of this model system is the ability to tune the domain size by varying the ratio of DOPC to POPC in the unsaturated *L*_d_ phase, with an increasing fraction of DOPC leading to larger rafts. Furthermore, there are regions in the phase diagram where the lipids are miscible. This allows for homogeneous, non-phase separating LUVs, which are used as a control and labeled as “no raft”. Using the phase compositions presented in the Supporting Information (SI) of ref (29), vesicles with a lipid composition matching that of the *L*_d_ and *L*_o_ phases of the large raft-forming LUVs, respectively, were prepared and investigated to asses potential phase dependent effects of peptide insertion. Additionally, single-lipid vesicles composed of DSPC, POPC, or DOPC, respectively, were separately investigated (data presented in the SI, section S5.). For all vesicles, a small fraction of 2.5 mol % DMPE-PEG was added to stabilize the vesicles against aggregation upon peptide addition, as well as to reduce the potential for multilamellarity of the vesicles, as established previously by Nielsen et al.^36^ The specific molar ratios of lipids in the different vesicle systems investigated are listed in Table 1.

**Table 1:** Nominal Molar Fractions of Lipids in the Vesicles Considered in This Study

vesicle	DSPC^a^	POPC	DOPC	cholesterol	DMPE-PEG
no raft	0.317 (78.2)	0.317	0	0.341	0.025
small	0.380 (67.4)	0.360	0.020	0.215	0.025
medium	0.380 (67.3)	0.321	0.059	0.215	0.025
large	0.380 (67.1)	0.243	0.137	0.215	0.025
large *L*_d_ phase	0.088	0.497	0.273	0.117	0.025
large *L*_o_ phase	0.536	0.117	0.059	0.263	0.025
DSPC	0.975	0	0	0	0.025
POPC	0	0.975	0	0	0.025
DOPC	0	0	0.975	0	0.025

a The percentage of tail-deuterated DSPC-d70 out of the total DSPC used for the SANS measurements is shown in parentheses.

All the vesicles were prepared using the same method: the desired amount of each lipid was weighed out and transferred to a round-bottom flask. Then, the dry lipid mixture was dissolved in a 1:3 methanol/chloroform solution to the same concentration as the final vesicle suspension. The organic solvent was evaporated completely using a Heidolph rotary evaporator with a Vacuubrand pump at a pressure of 40 mbar and at a temperature a few degrees above the melting temperature of the lipid in the mixture with the highest melting temperature. The resulting lipid film was hydrated in 50 mmol of Tris buffer of pH = 7.4 for 1 h followed by sonication for 20 min at the same temperature as above. Finally, the vesicle suspension was extruded through a polycarbonate filter with a 100 nm pore diameter at least 25 times using an Avanti mini-extruder, resulting in unilamellar and relatively monodisperse vesicles with an average diameter close to 100 nm. These vesicles are commonly termed large unilamellar vesicles (LUVs) and are believed to be sufficiently large to not exhibit any curvature dependent effects.^38^ The vesicles were prepared as close as possible to the experiment and only briefly stored at 5 °C.

The antimicrobial peptides indolicidin and LL-37 were purchased from TAG Copenhagen A/S and used as received, while aurein 2.2 and magainin II were purchased from Schafen-N ApS, Copenhagen, and used as is. With the exception of LL-37, the peptides were added to the vesicles in a 1:20 peptide:lipid (PL) molar ratio. LL-37 was added in a substantially lower 1:100 PL ratio, as it is known to cause solubilization at higher PL ratios in DMPC/DMPG membranes.^18^ In the case of SAXS, indolicidin was also added in a higher (1:10) and a lower (1:50) PL ratio. Stock solutions were prepared by dissolving the peptides in the same Tris buffer as was used for the vesicles. Diluted peptide solutions were prepared from the stock such that mixing the peptide and vesicle solutions in 1:1 volume ratios would result in the desired PL ratios. The peptide and vesicles were mixed and incubated for approximately 30 min prior to measurement.

### Isotope Labeling and Contrast Variation in SANS

**Figure 1:**
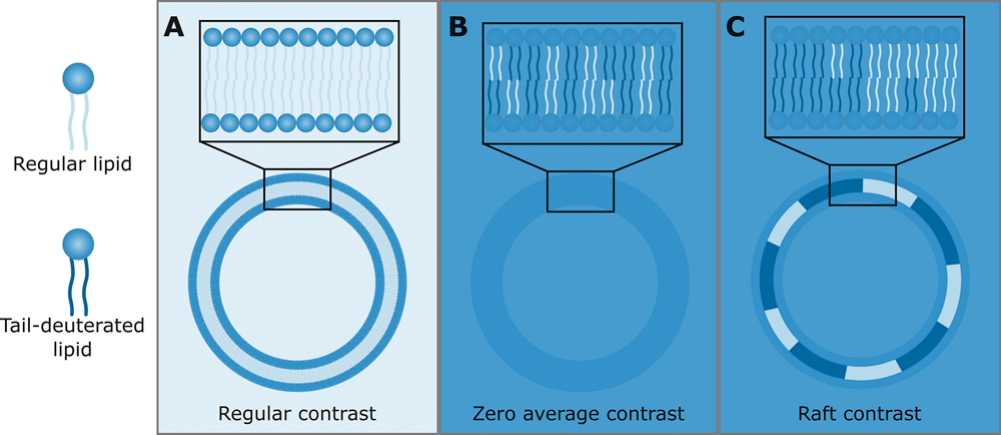
Illustration of the different contrasts achievable with SANS and the use of tail-deuterated lipids; a darker shade of blue represents higher neutron SLD. (A) The contrast seen for a liposome made up of only regular (nondeuterated) lipids in H_2_O. (B) Zero average contrast, where tail-deuterated and regular lipids are mixed in a specific ratio so that the average SLD of the tails matches the heads. The solvent is a mixture of H_2_O and D_2_O with SLD also matching the head groups. (C) Phase separation into one phase rich in tail-deuterated lipids and a phase rich in nondeuterated lipids, as is the case for rafts, results in strong lateral contrast.

In order to visualize the lipid rafts using SANS, a zero average contrast (ZAC) technique was used, which utilizes the difference in coherent neutron scattering lengths of hydrogen (H) and deuterium (D), respectively. With protiated phospholipids, the head group will have a higher scattering length density (SLD) compared to the tail group, which results in a contrast between the two head groups and the tails in a bilayer (Figure 1A). For tail-deuterated lipids, the hydrogen in the acyl tail chains is replaced with deuterium, while the head group remains the same. In this case, the tails will have a higher SLD than the heads, resulting in a contrast with opposite sign between the two. At a specific ratio of regular and tail-deuterated lipids, the average SLD of the tails will match that of the head groups. Furthermore, the solvent SLD can also be matched to the head group SLD by using an appropriate mixture of H_2_O and D_2_O. The result is a zero average contrast, as shown in Figure 1B.

**Figure 2:**
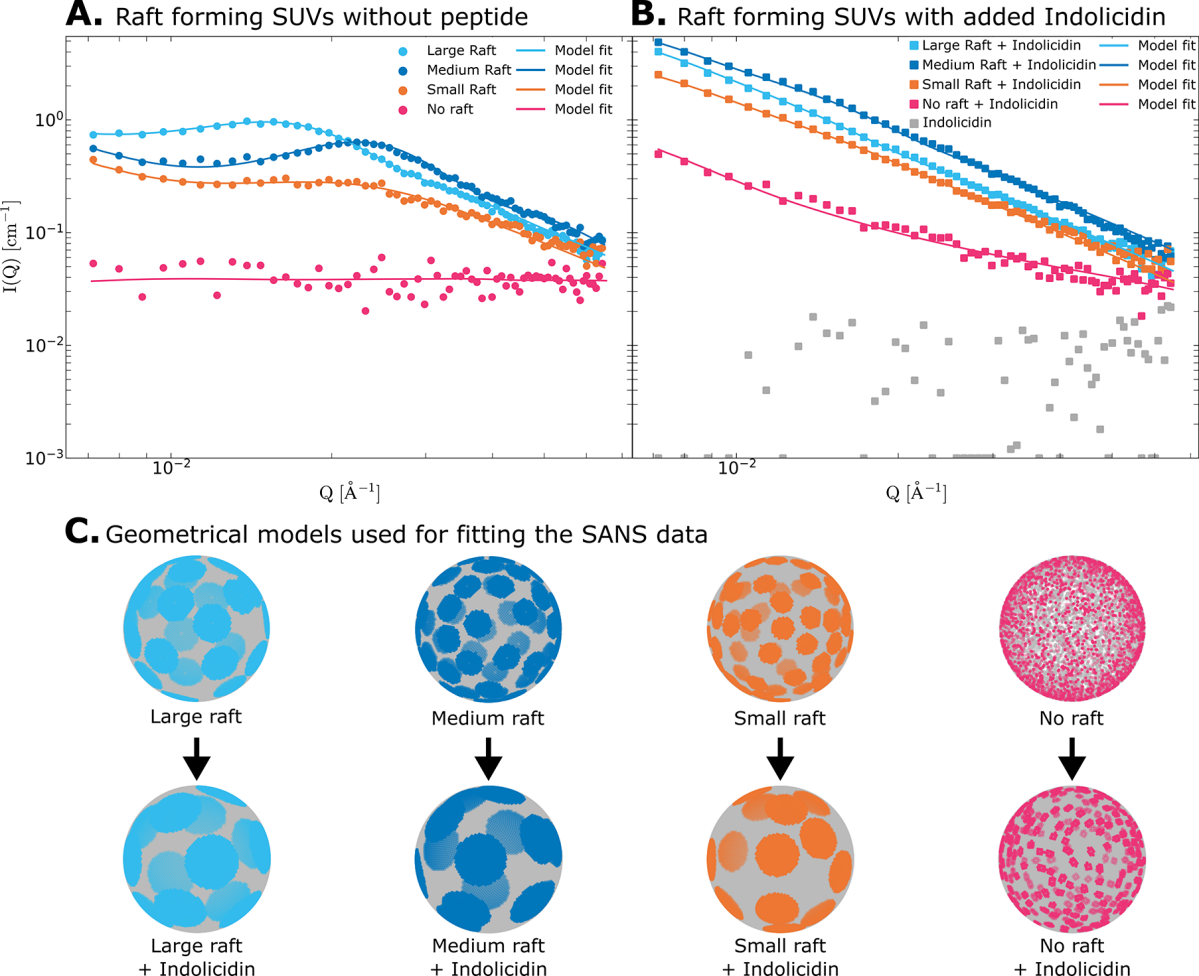
SANS scattering curves on a log–log scale for the three raft-forming LUVs and the no raft control LUV (A) without peptide and (B) with indolicidin added in a 1:20 peptide:lipid molar ratio. Model fits are shown as solid lines and correspond to the geometrical models shown in (C) (top: without peptide; bottom: with peptide added).

This technique can be used to visualize rafts in the following way. A specific fraction of the saturated DSPC is replaced with tail-deuterated DSPC-d70 (percentage of DSPC-d70 relative to the total amount of DSPC is shown in parentheses in Table 1), and a fraction of H_2_O in the solvent is replaced with D_2_O so that the average neutron scattering length density (SLD) of both the solvent and lipid tails matches the SLD of the lipid heads, which in this case is 0.185 fm/Å^3^. This is achieved by using a 34.8% D_2_O mixture (accounting for the scattering contribution of 0.050 M Tris). In the absence of phase separation, the protiated and tail-deuterated lipids are homogeneously mixed, and the LUVs are contrast matched with the solvent (Figure 1B). This results in essentially flat scattering, as is observed for the non-raft-forming control (magenta) in Figure 2A. When the membrane phase separates and rafts are formed, there is a lateral segregation between the protiated and deuterated lipids, which will predominantly be in the DSPC rich *L*_o_ phase. This results in high contrast between the two phases, as shown in Figure 1C, and the scattering curves for the raft-forming LUVs (Figure 2A) display a characteristic peak related to the size, number, and spatial distribution of the lipid rafts.

### Small-Angle Neutron Scattering

The small-angle neutron scattering (SANS) measurements of the isotope labeled vesicles (data shown in Figures 2 and 7) were performed at the SANS-1 beamline at the SINQ spallation neutron source at Paul Scherrer Institut (PSI), Villigen, Switzerland. A sample to detector distance of 8 m was used, resulting in a *Q* range approximately from 6.31 × 10^–3^ to 6.42 × 10^–2^ Å^–1^, using a neutron wavelength of λ = 7.00 Å and a wavelength resolution of Δλ/λ = 0.1. The samples were placed in 1 mm Hellma quartz cuvettes and mounted in a temperature controlled sample stage kept at 20 °C. Buffer was separately measured and used for background subtraction. Sample transmissions, empty beam, blocked beam, and water standard measurements were used to scale the sample scattering to an absolute scale. Data reduction and radial averaging were performed using the BerSANS software package. For all SANS measurements, a liposome concentration of 10 mg/mL was used.

**Figure 3:**
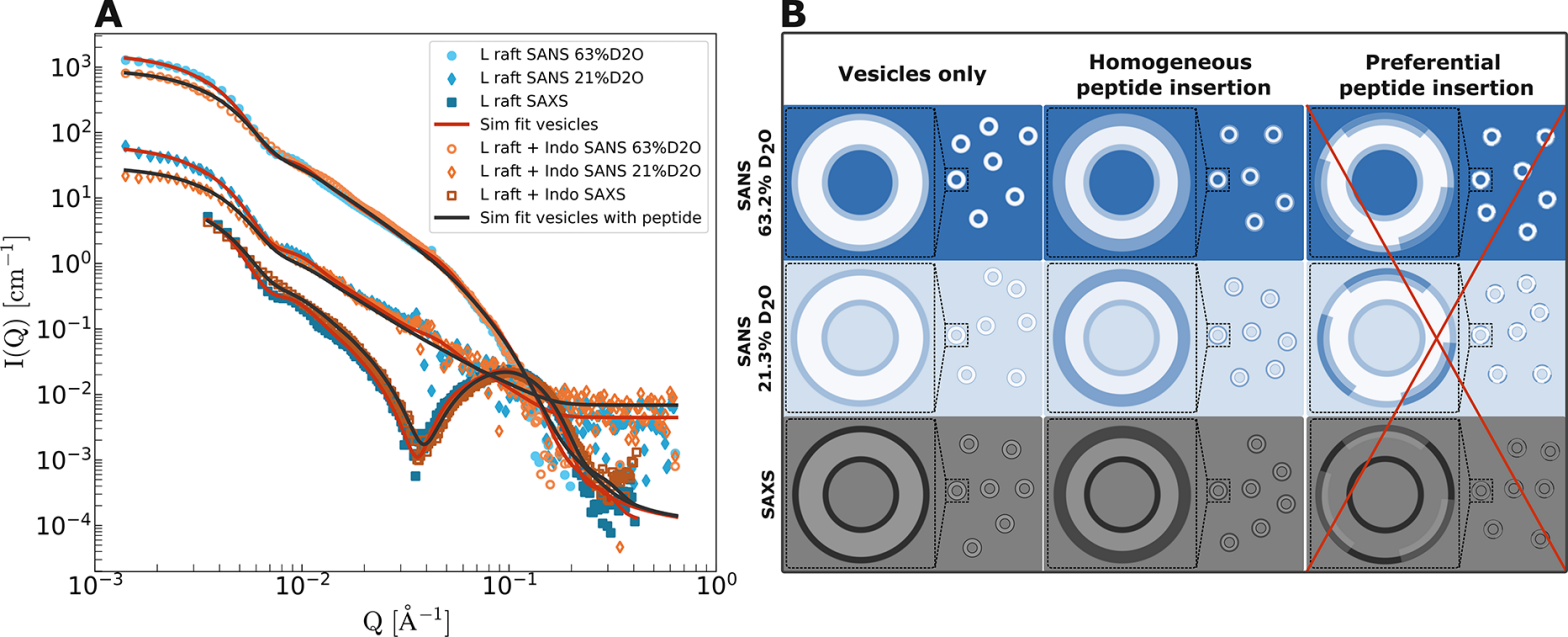
(A) SAXS (squares) and SANS curves with 21.3% (diamonds) and 63.2% (circles) D_2_O of tail protiated vesicles forming large rafts without peptide (blue filled data points) and with indolicidin added in a 1:20 PL ratio (orange empty data points). Solid lines are obtained by simultaneous fitting of the three-shell analytical model to the three separate contrasts; red solid lines for vesicles without peptide and black solid lines for vesicles with indolicidin. All curves are at absolute scale. (B) Illustration of the contrast conditions that give rise to the scattering curves shown in (A) for pure vesicles (left panes) and homogeneous peptide insertion (middle panes). The lateral contrast that would arise from preferential peptide insertion (right panes) is not observed in the experimental scattering curves.

Additional SANS measurements of large raft-forming vesicles (Figure 3) were performed at the D22 beamline at Institut Laue-Langevin (ILL) in Grenoble, France (experiment DOI: 10.5291/ILL-DATA.EASY-1301). The simultaneous use of two detectors at 1.40 and 17.6 m, respectively, and merging of repeated measurements at two neutron wavelengths of 6.0 and 11.5 Å, both with Δλ/λ = 0.1, resulted in an extensive *Q* range from 1.40 × 10^–3^ to 6.44 × 10^–1^ Å^–1^. Samples were measured at 20 °C in 1 mm Hellma quartz cuvettes with a concentration of 10 mg/mL, and the resulting scattering intensity was calibrated to absolute scale. These vesicles were composed of only tail protiated lipids but with the same raft-forming lipid composition as was used for the large rafts shown in Table 1. The vesicles were measured in 21.3% and 63.2% D_2_O buffer to match the average contrast between the lipid heads and protiated tails and to provide a good overall contrast, respectively.

### Small-Angle X-ray Scattering

**Figure 4:**
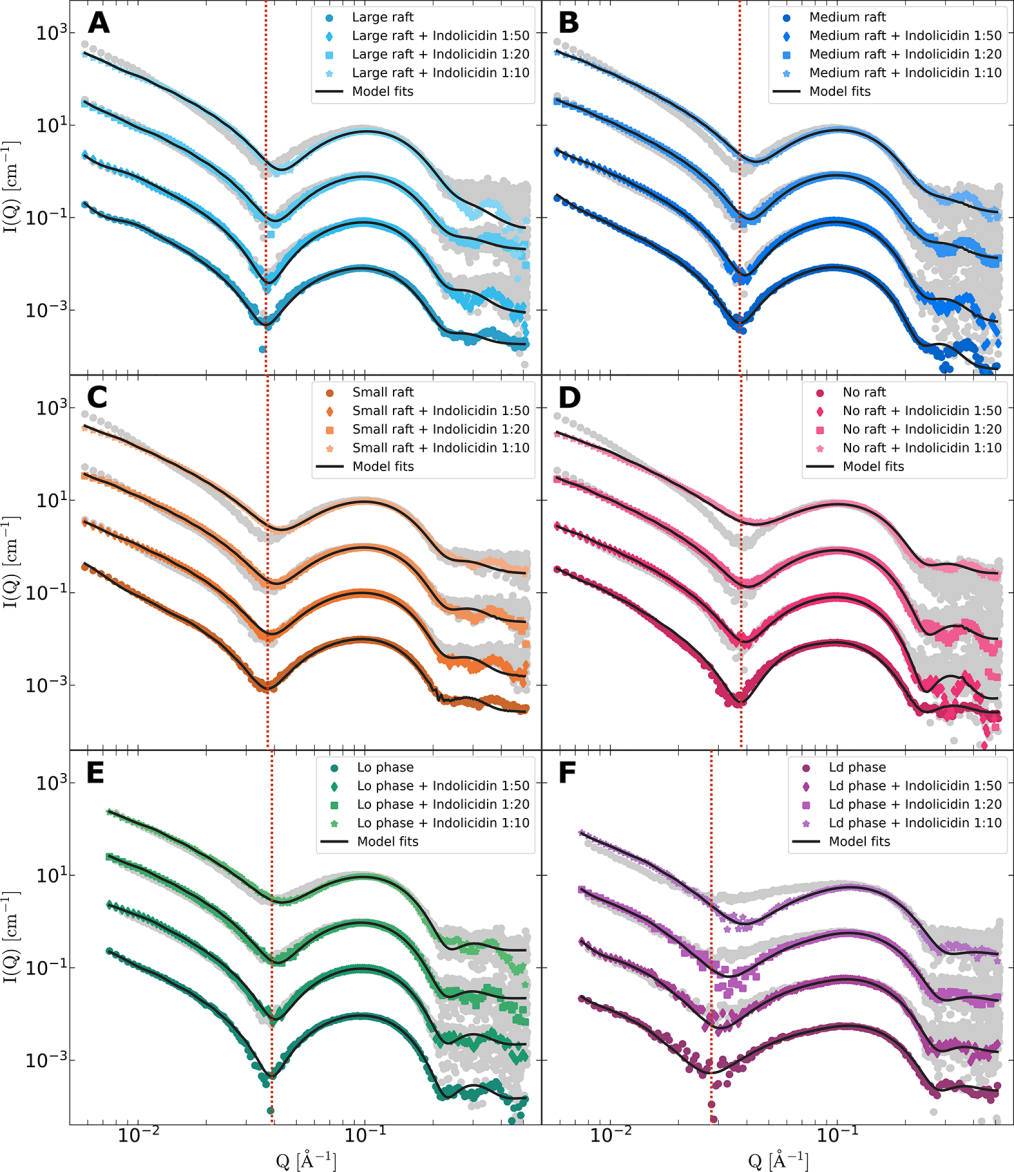
SAXS scattering curves of vesicles forming (A) large rafts, (B) medium rafts, (C) small rafts, and (D) no rafts, as well as the separate (E) *L*_o_ and (F) *L*_d_ phases, with increasing amount of indolicidin added. The bottom scattering curve in each panel (A–F) shows the liposome scattering with no added peptide at true scale. In ascending order, the curves show liposomes with the peptide added in 1:50, 1:20, and 1:10 PL ratios and are multiplied by 10, 100, and 1000, respectively, for better visualization. Model fits are shown by the solid lines, while the calculated averages, i.e., if there are no interactions between vesicle and peptide, are shown with gray scattering points. A red dotted line is added to each panel to highlight the shift in the minima.

The majority of small-angle X-ray scattering (SAXS) experiments (data shown in Figures 4 and 7) were performed at the BM29 BioSAXS beamline at the European Synchrotron Radiation Facility (ESRF) in Grenoble, France.^39^ A beam energy of 12.5 keV and 100% transmission were used with an experimental *Q* range from 5.31 × 10^–3^ to 5.21 × 10^–1^ Å^–1^. The automatic sample changer of the instrument was used to flow 50 μL of the sample solution at a constant flow rate through a quartz capillary as 10 successive frames with 1 s exposure time were automatically collected and reduced to one-dimensional *I*(*Q*) curves, set to absolute scale using water as a standard. Frames showing indications of radiation damage were excluded, and the remaining frames averaged. Buffer was measured before and after each sample, and the average buffer scattering was subtracted from the sample scattering. SAXS measurements were done at 20 °C with a liposome concentration of 2.5 mg/mL.

Additional SAXS experiments were performed at the ID02 beamline^40^ at ESRF in Grenoble, France. An energy of 12.23 keV and a sample detector distance of 2.0 m were used in combination with a 2 mm flow-through cell. This resulted in an experimental *Q* range from 3.51 × 10^–3^ to 4.13 × 10^–1^ Å^–1^. In this case, the exact same large raft-forming vesicles prepared for the SANS experiments at D22 were used and measured at the same concentration of 10 mg/mL with and without the addition of indolicidin at a 1:20 PL ratio at 20 °C. Ten successive frames of 0.01 s exposure time were set to absolute scale and averaged together for each sample, followed by buffer subtraction.

### Differential Scanning Calorimetry

The samples were investigated using differential scanning calorimetry (DSC), specifically with a Nano DSC instrument from TA Instruments. Measurements were performed with a scan rate of 2 °C/min from 15 to 75 °C. The buffer was measured at the same settings and subtracted from the thermograms using the NanoAnalyze software from TA Instruments. Using the same software, the baseline was found and subtracted, and the data were converted to specific heat capacity, *C*_p_, in kJ mol^–1^ K^–1^.

### Modeling the Scattering Intensity

Due to the fundamental difference in contrast, two different models were developed and used to respectively describe the data from the isotope labeled vesicles to study the lipid rafts and the SANS/SAXS data that renders the overall structure visible. The ZAC SANS measurements with selective isotope labeling are mostly sensitive to the lateral, in-plane contrast between the two phases and primarily contain information about the size and distribution of the rafts. In order to describe the experimental scattering intensity, a discrete model is used in this case. Here the raft-forming vesicles are described by a large number of small spherical beads, which are distributed on the surface of a sphere with a radius corresponding to that of the vesicles. Each bead is labeled as belonging either to the rafts or to the continuous phase with their respective SLDs. The total domain area, which is given from the lipid composition, is divided into *N*_dom_ circular domains. These domains are nontouching but are otherwise randomly distributed on the vesicle surface. The total scattering intensity is then computed as a sum of the contribution from each pair of individual beads by using the Debye equation. The complete SANS model, which also incorporates polydispersity in size and instrumental resolution, is described in detail in the SI, section S1.

In the case of SAXS, the contrast arises from the difference in electron density (ED) in different parts of the sample. For vesicles in solution, the primary difference in ED is between the lipid heads and the lipid tails relative to the aqueous buffer. Thus, the scattering intensity is mainly dependent on the radial contrast, i.e., transversal to the bilayer plane, and contains information on the size and size distribution of the vesicle in addition to the bilayer thickness and asymmetry. SAXS is therefore very sensitive to peptide insertion and position in the bilayer. The SAXS and SANS data on fully protiated lipid vesicles were analyzed simultaneously using a concentric shells model, where three concentric spherical shells are used to describe the inner lipid head groups, the hydrocarbon tails, and the outer head groups, respectively. An overview of this model is presented in a recent review by Nielsen et al.^41^ However, several additional considerations were necessary in order to adapt the initial model to the present system. These include accounting for the significant cholesterol fraction and the difference in the bilayer thickness of the two coexisting phases. A complete and detailed description of this model is found in the SI, section S2.

## Results and Discussion

### Peptide Insertion Causes Domain Growth

We employed the well-known raft-forming model system consisting of a quaternary lipid mixture of DSPC, POPC, DOPC, and cholesterol to prepare four different LUV samples as described above. Three of these form small, medium, and large nanoscale rafts, respectively, while the last is a non-raft-forming control. Figure 2 shows the SANS data from the three raft-forming LUVs and the non-raft-forming control, freely floating in aqueous solution at 20 °C and physiological pH, without peptide (Figure 2A) and with indolicidin added in a 1:20 peptide:lipid (PL) molar ratio (Figure 2B). The lipid rafts are visualized by enhancing the lateral contrast between the two phases through isotope labeling and contrast matching, as previously described. The SANS curves of the LUVs without the addition of peptide feature a distinct peak at intermediate *Q*. The position and shape of this peak is largely determined by the number, average size, and spatial distribution of the rafts, and the experimental data are analyzed using the geometrical model described above (solid lines). The resulting structures are shown in Figure 2C, while the fitted model parameters are shown in Table 2.

**Table 2:** Vesicle Radius, Number of Domains, and Domain Area for the Raft-Forming LUVs Based on SANS Model Fits

sample	LUV radius [nm]	number of domains	domain area [nm^2^]^a^
large	63	26	825
medium	60	50	380
small	68	56	321
no raft	60	na	na
large + indolicidin	65	13	1500 (19 450)
medium + indolicidin	60	14	1360 (19 000)
small + indolicidin	68	15	1200 (18 010)
no raft + indolicidin	60	250	36
large^b^	60	29	686
large^b^ + LL-37	68	22	1162 (25 600)
large^b^ + magainin II	68	20	1280 (25 600)
large^b^ + aurein 2.2	65	12	2130 (25 600)

a Scattering of the raft-forming LUVs with added peptide is fitted with a coexistence model of one and several domains; area of the single domain is shown in parentheses.

b This is a second batch of LUVs prepared and measured at a different time compared to the first large raft LUV batch.

The results for the raft-forming LUVs without peptide (Figure 2A) are in excellent agreement with the results presented by Heberle et al.,^29^ where a similar raft structure is reported, although it is worth mentioning that in the present case, vesicles with ∼100 nm diameter were employed as opposed to the ∼50 nm ones used by Heberle et al.^29^ This relatively large difference in membrane curvature as well as small variations in the lipid composition hinder a direct one-to-one comparison. As expected, the scattering from the homogeneous non-raft-forming sample (magenta) is flat and well described by a vesicle where the lipids are randomly mixed (Figure 2C).

To investigate the effect of AMP on the lateral phase separation, indolicidin was added in a 1:20 PL molar ratio to the three raft-forming LUVs as well as the non-raft-forming control. The resulting SANS curves with corresponding model fits are shown in Figure 2B,C. Interestingly, the addition of the peptide causes a large increase in the scattering intensity for the raft-forming LUVs, particularly at low to intermediate *Q*.

The increase in intensity cannot trivially be attributed to the simple addition of the extra peptide scattering, as the scattering from the peptide by itself (gray squares in Figure 2B) is essentially flat and barely above the background due to the low contrast and concentration. On the other hand, one might imagine that the peptide clusters or assembles on the vesicle, creating larger structures. However, we need to consider the contrast conditions. By experimental design, the average SLD of the lipid tails matches that of the lipid heads, which are also matched by the buffer and essentially match that of the peptide. The SLD of the peptide is 0.241 fm/Å^3^, while that of the buffer is 0.185 fm/Å^3^. Moreover, upon phase separation, the *L*_o_ phase will contain the majority of tail-deuterated lipids and have a higher SLD, while the *L*_d_ phase will have a lower SLD, 0.275 and 0.027 fm/Å^3^, respectively. Thus, preferential insertion of the peptide into the *L*_d_ phase would bring the average SLD of that phase closer together and lower the overall contrast. On the other hand, preferential partitioning into the *L*_o_ phase would also lower the contrast since the average SLD would decrease. Nevertheless, we performed detailed calculations confirming the qualitative considerations in the SI, section S1.4.

The observed increase in intensity can only be explained by a significant increase in the raft size. The clustering of d-lipids causes a substantial change in contrast, which results in the most significant increase in intensity. Model fits reveal much fewer and significantly larger domains (Figure 2C), while the total area fraction of each phase remains constant, suggesting that the lipid rafts grow due to coalescence upon peptide addition.

Unlike the LUVs without peptide, the scattering from the LUVs with added peptide cannot be completely described by a single number of domains *N*_dom_, but instead displays some heterogeneity. To explain the experimental data, a coexistence model of LUVs with a single domain (*N*_dom_ = 1) and LUVs with a few very large domains (*N*_dom_ = 15, 14, and 13 for the small, medium, and large rafts, respectively) was used. Notably, the scattering curves with added peptide are remarkably similar to the scattering curves reported for the very large rafts (the D7 composition) by Heberle et al.^29^ In this case, the authors also used a coexistence model of *N*_dom_ = 1 and *N*_dom_ = 4 to explain the very large rafts. This might indicate that the system is highly dynamic, where the shape and size of the domains fluctuate, e.g., by a constant coalescence and dissociation process. Due to the finite size, a large fraction of the LUVs seems to be completely phase separated, forming “Janus”-like vesicles. This is well documented in GUVs with large line tension and where DOPC is used as the unsaturated lipid.^25,28^ See the SI, section S1.5 for a further discussion of the coexistence model.

Furthermore, it is interesting that the scattering intensity also increases when the peptide is added to the non-raft-forming control sample. This indicates the formation of domains upon peptide addition, and the data can be described well by using a model with a large number (*N*_dom_ = 250) of small domains. This suggests that the peptide not only causes growth of the rafts in already raft-forming membranes but may also induce phase separation in an initially laterally homogeneous, nonsegregated membrane.

### The Peptides Partition into Both Phases and Cause Differential Membrane Thinning

From the SANS data, it becomes clear that the addition of indolicidin to raft-forming vesicles causes significant raft growth and lateral reorganization of the membranes. However, it is not directly apparent how and where the peptide inserts, nor if it causes any structural changes to the bilayer. Indications that AMPs can have a preference for specific lipid phases are seen from the dye leakage assays by McHenry et al.,^22^ which show that saturated, unsaturated, and phase separated membranes are lysed differently by certain peptides. Additionally, the comprehensive simulations by Su et al.^42^ suggest that certain AMPs, including magainin II, which is also considered here, have a preference for the *L*_d_ phase composed of the highly unsaturated dilinoleoyl phosphatidylcholine (DLiPC) and cholesterol, as opposed to the *L*_o_ phase of saturated dipalmitoyl phosphatidylcholine (DPPC) and cholesterol.

In order to get more insight into the peptide insertion and the resulting domain growth, we need to investigate any preferential partitioning of the peptide into the two phases. Since the contrast conditions for the labeled vesicles do not allow us to determine the lateral peptide distribution in detail, we designed another set of experiments. Here a single batch of LUVs forming large rafts was prepared by using fully protiated lipids and exposed to indolicidin. We then used two different contrasts in SANS as well as synchrotron SAXS to study the peptide vesicle interactions at the same concentration and temperature (Figure 3). Crucially, all lipids were now protiated, resulting in a neutron scattering signal dominated by the radial (i.e., transversal to the bilayer plane) contrast arising from the difference in scattering length between the lipid heads and tails. Additionally and unlike neutrons, SAXS is sensitive to the differences in electron density (ED). As this difference in ED is particularly large between the lipid heads and tails, the contrast is also in this case mainly radical and not lateral. Furthermore, to resolve the peptide distribution in more detail, we also employed a “weak” contrast condition where the average lipid scattering is matched using a 21.3% D_2_O buffer solution. Here the SLD matches the average between the heads and tails, rendering the lipid vesicles less visible. Therefore, any “excess” scattering from the peptide due to structuring or any deviation from a spherical shell type scattering can then be detected. These contrast conditions are illustrated in Figure 3B.

Very interestingly, the data indicate that the peptides are evenly distributed. As seen in Figure 3, the scattering curves resulting from the three contrasts can all be nicely described by the regular radially symmetric three-shell model, both with and without peptide (black and red solid lines, respectively). The data analysis was performed simultaneously using all three contrasts (SAXS and SANS), and it clearly shows that no excess scattering from potential peptide clustering (illustrated in the right pane of Figure 3B) was observed. This strongly suggests that the peptide distributes more uniformly in both phases. Furthermore, the analysis reveals that the peptide is primarily in the outer bilayer leaflet, and we see that upon peptide addition, the average hydrocarbon thickness of the bilayer reduces from approximately 27 to 23 Å, while the standard deviation of the thickness polydispersity increases from 0.18 to 0.25. A more detailed discussion, including all resulting fit parameters, is reported in the SI, Table S4.

Although it might initially seem so, it is perhaps not so surprising that the peptide can insert seemingly uniformly into both phases. For the quaternary lipid mixture considered here, the phase composition is not trivial, as demonstrated by the extensive work of Heberle et al.^29^ needed to map the four-dimensional phase space. They show that each phase contains significant molar fractions of all four lipids (see the SI of ref (29)), making the two phases somewhat similar. This is markedly different to the lipid composition considered in the work of Su et al.,^42^ where the large difference in saturation between polyunsaturated DLiPC and saturated DPPC seemingly results in nearly complete lipid separation between the two phases. This, in addition to the different AMPs considered here, might account for the difference in peptide partitioning observed in the present work. Nonetheless, the effects of lipid composition and phase separation on peptide partitioning pose interesting questions for further studies.

To further characterize the effects on the bilayer structure caused by indolicidin, a systematic small-angle X-ray scattering (SAXS) study was carried out. Here LUVs with the same raft-forming lipid compositions were measured with and without the addition of indolicidin. In addition to the 1:20 PL ratio, higher (1:10) and lower (1:50) PL ratios were also measured in order to discern any potential concentration dependent effects. Additionally, LUVs with a lipid composition matching those of the *L*_d_ and *L*_o_ phases of the large raft-forming vesicles were measured. The SAXS curves, including model fits, are shown in Figure 4.

The SAXS data in Figure 4 show the typical form factor expected for lipid vesicles.^43^ It is clearly seen that with the addition of peptide, the minima is shifted toward higher *Q*. Additionally, the low *Q* slope is affected, as can be seen from the difference between the experimental scattering curves and the underlying calculated average (gray data points). This is the expected average scattering from the separate peptide and vesicle scattering in the case of no interactions between the two. These changes are consistent with peptide insertion into the bilayer and resulting contrast changes, as reported previously by Nielsen et al.^18^ The analysis reveals that the peptide is primarily located in the outer leaflet at the concentrations considered. This is consistent with the results reported for model membranes consisting of 1,2-dimyristoyl-*sn*-glycero-3-phosphocholine (DMPC) and 1,2-dimyristoyl-*sn*-glycero-3-phospho-(1′-*rac*-glycerol) (DMPG) based on SAXS^36^ and neutron reflectometry (NR).^35^ For all vesicle compositions and PL ratios, the experimental data is well described without any free peptide fraction, i.e., all peptide is assumed to be partitioned into the vesicles. Details are given in the SI.

**Figure 5:**
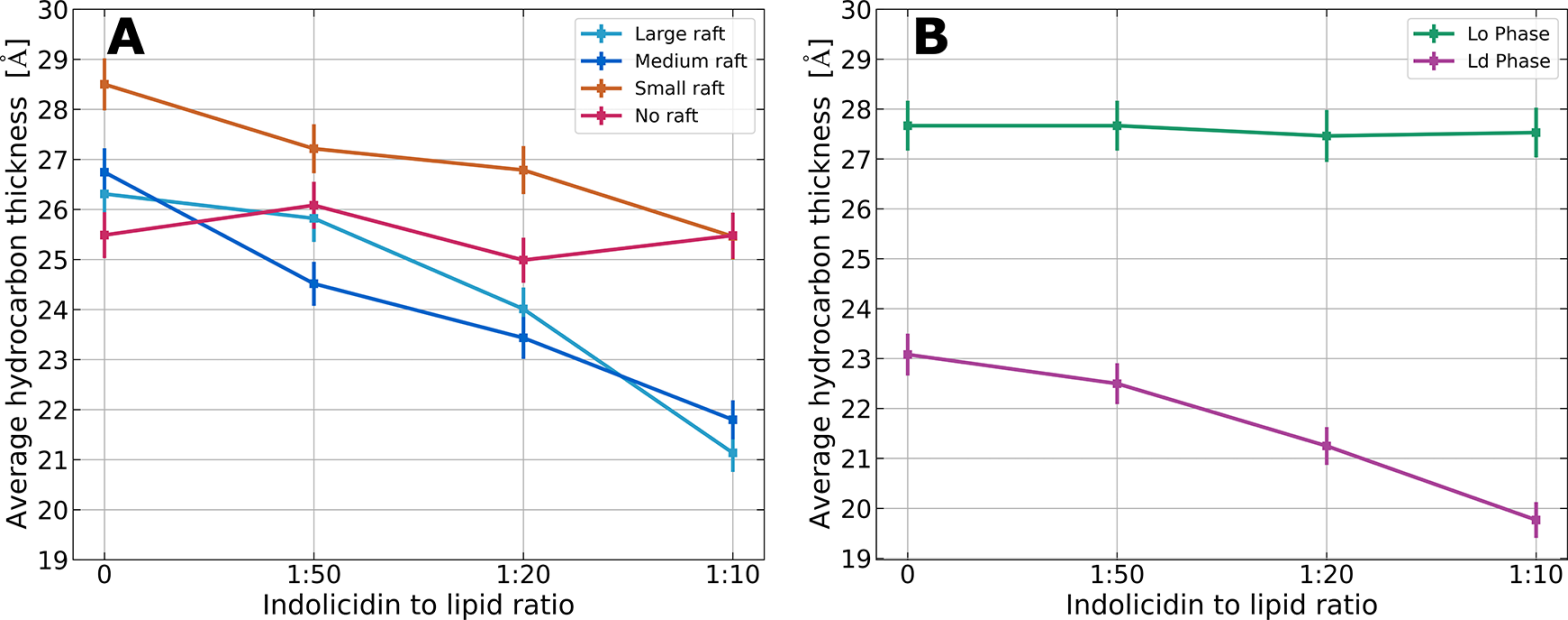
Hydrocarbon thickness as a function of peptide ratio for (A) the raft-forming vesicles and (B) the separate *L*_o_ and *L*_d_ phases resulting from the model analysis of the scattering curves presented in Figure 4. Colors correspond across the figures.

In the case of raft-forming vesicles (Figure 4A–C), the addition of peptide systematically reduces the average thickness of the bilayer hydrocarbon region as the peptide concentration increases. This reduction is not observed for the non-raft-forming control (Figure 4D). Interestingly, the separate *L*_o_ and *L*_d_ phases show distinct effects of peptide addition on bilayer thickness, as seen in Figure 5B. While the thickness of the saturated *L*_o_ phase is largely unaffected, the unsaturated *L*_d_ phase experiences a systematic decrease in thickness from approximately 23 to 20 Å. Similar effects are observed for single-lipid membranes composed of DSPC, POPC, or DOPC, as detailed in the Supporting Information. Additionally, a decrease in the average bilayer thickness and a systematic increase in the polydispersity of bilayer thickness are observed for the raft-forming vesicles (see the SI, section S2.1). In summary, the SAXS and simultaneous SAXS/SANS results indicate that the peptide preferentially reduces the thickness of the *L*_d_ phase, thereby increasing the thickness difference between the two phases.

It bears mentioning that although the three-shell model accurately and robustly explains a wide range of contrasts and length scales in combined SAXS and SANS analyses (Figure 3), it does not fully capture the high-*Q* secondary oscillations observed in the SAXS data. These oscillations, more pronounced in membranes with high lipid order such as *L*_o_ phase membranes (Figure 4E), arise from very short-range correlations. An alternative approach, the scattering density profile (SDP) model,^44^ which describes each component of the bilayer with a separate Gaussian function, could potentially provide better fits at high *Q*.^41^ However, implementing this model would considerably increase the complexity and number of fitting parameters, especially given the numerous components in the membranes studied. Considering the good fits achieved with significantly fewer parameters in the three-shell model, the use of the SDP model was not justified.

### Peptide Addition Increases Phase Transition Temperature and Stabilizes *L*_o_ Phase

**Figure 6:**
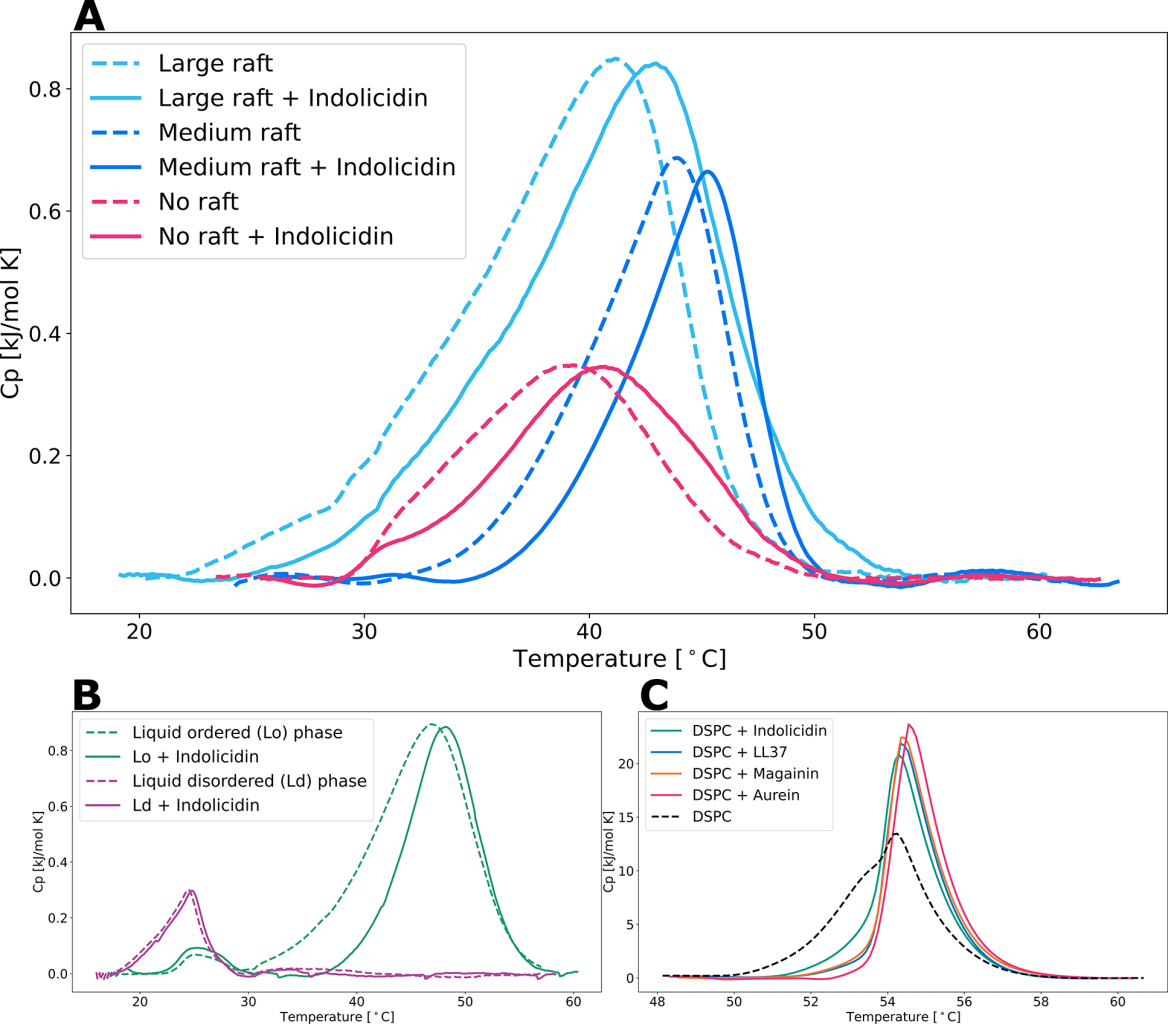
DSC heating curves, with and without indolicidin in a PL ratio of 1:20, of (A) raft-forming samples with differently sized domains, (B) liquid ordered and disordered phases, and (C) pure DSPC vesicles with the additional peptides LL37 in a 1:100 ratio and magainin II and aurein 2.2 both in 1:20 PL ratios.

In addition to the small-angle scattering experiments, differential scanning calorimetry (DSC) was also used to investigate the peptide–vesicle interactions. As shown in Figure 6A, the addition of indolicidin to the raft-forming samples leads to a significant increase in the phase transition temperature (*T*_m_). Due to the broad transitions observed and difficulties in baseline determination, the accurate determination of Δ*H* is challenging. Nevertheless, the clear temperature shift is consistent with a higher degree of phase separation, as observed in the SANS results. Similarly, the separate *L*_d_ and *L*_o_ phases (Figure 6B) also exhibit higher *T*_m_ values upon peptide addition. While the shift is slight for the *L*_d_ phase, the *L*_o_ phase shows a comparable increase in *T*_m_ and a noticeable sharpening of the phase transition peak. Hence, the thermograms suggest that indolicidin not only interacts with the *L*_o_ phase but also has an ordering effect, likely stabilizing the ordered lipid structure. This ordering effect and increase in *T*_m_ are particularly evident in the pure DSPC membrane (Figure 6C), where other natural AMPs such as LL-37, magainin II, and aurein 2.2 also exhibit similar ordering and *T*_m_ shifts.

The observed increase in *T*_m_ is consistent with an increase in line tension and a higher degree of phase separation. If the peptide instead were acting as a lineactant and lowered the line tension, we would expect a smaller *T*_m_ and broadening of the peak. The change in cooperation in the transition is strongest in the DSPC vesicles, which may be due to finite size effects. We speculate that the observed effects might be due to the peptide binding to the head group at the interface, increasing the lateral organization of the lipids. One might speculate that the peptide also induces changes in the phase composition, e.g., of cholesterol. However, the same increase in *T*_m_ is observed in the “pure” DSPC vesicles, indicating an intrinsic peptide-binding effect.

### A Generic Phenomenon: AMPs Induce Domain Growth

**Figure 7:**
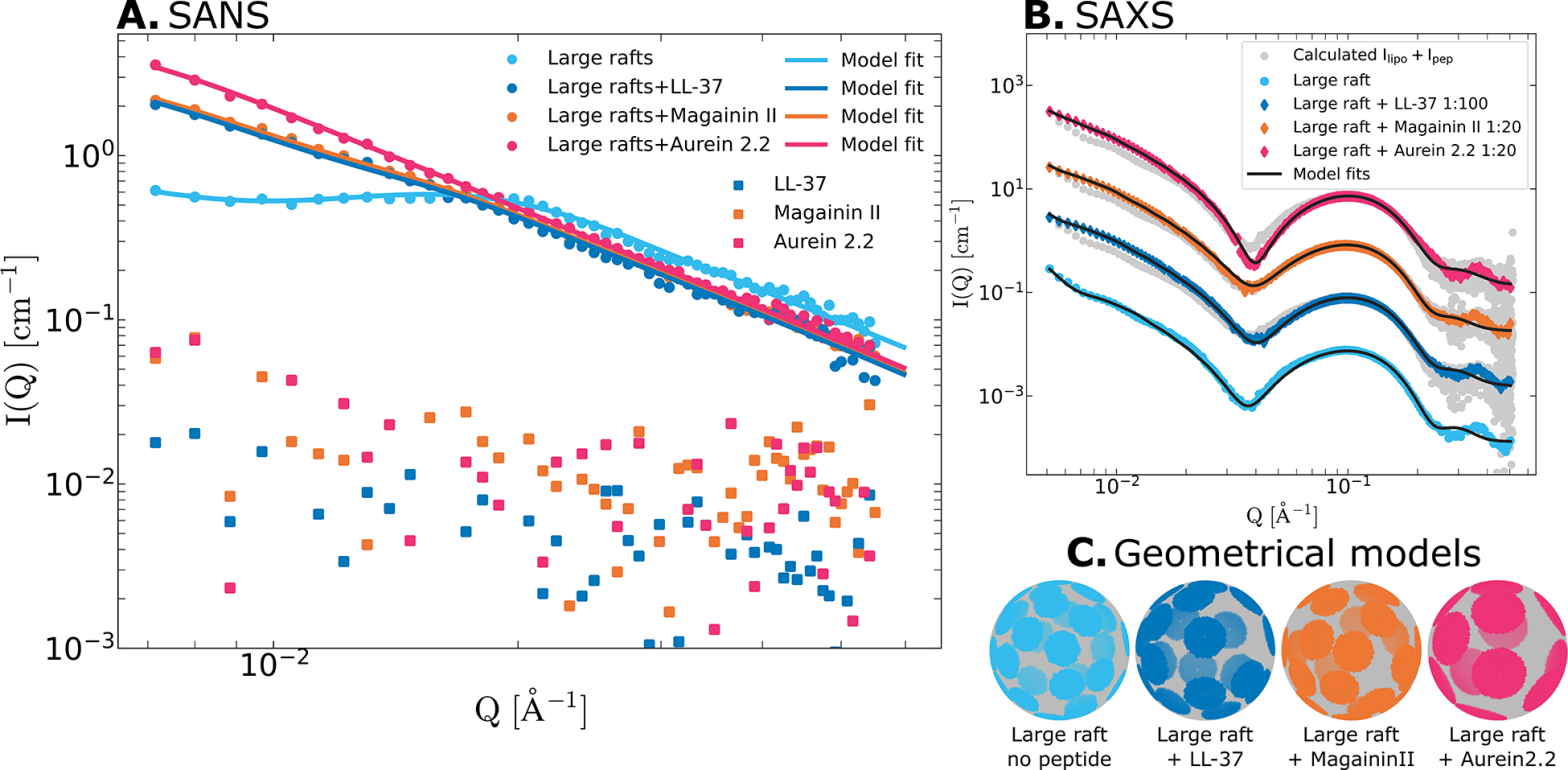
(A) SANS and (B) SAXS curves for LUVs forming large rafts without peptide (light blue) and with the addition of several different AMPs: LL-37 in a 1:100 PL ratio (dark blue), magainin II in a 1:20 PL ratio (orange), and aurein 2.2 in a 1:20 PL ratio (pink). (C) The geometrical models used to describe the SANS data; the colors correspond across the panels. Additionally, the SANS curves from the peptides by themselves are shown with squares in (A), and the calculated averages for the SAXS data (i.e., if there were no interactions between the LUVs and peptides) are shown in gray in (B).

In addition to indolicidin, several other natural antimicrobial peptides (AMPs) were investigated. The LUVs forming large rafts were exposed to LL-37, magainin II, and aurein 2.2 and characterized using both SANS and SAXS, as shown in Figure 7A,B. The selected peptides are all α-helical, as opposed to unstructured indolicidin. However, they differ greatly in origin, length, and net charge, and they represent a diverse set of natural AMPs. Interestingly, and despite the differences, all of the investigated peptides result in a similar, substantial growth of the lipid raft domains, although to various degrees. The resulting raft structures obtained from the analysis of the SANS data are depicted in Figure 7C, and the details (number of rafts and their sizes) are shown in Table 2. Similarly to indolicidin, the SANS scattering intensity greatly increases at low *Q* when the peptides are added. This can again not be explained simply with the added peptide scattering (squares in Figure 7A) and indicates the formation of larger structures. The SAXS scattering curves reveal that the peptide inserts into the bilayer but does not cause solubilization. Additionally, the SAXS model analysis again shows a reduction in the hydrocarbon shell thickness, followed by an increase in the polydispersity (model fit parameters shown in the SI, Table S10). The raft growth caused by LL-37, magainin II, and aurein 2.2, similarly to indolicidin, corresponds to increased interfacial line tension. Furthermore, the thinning and increased heterogeneity of the hydrocarbon shell suggest a similar mechanism for these AMPs as for indolicidin.

### Increased Line Tension through Differential Bilayer Thinning

**Figure 8:**
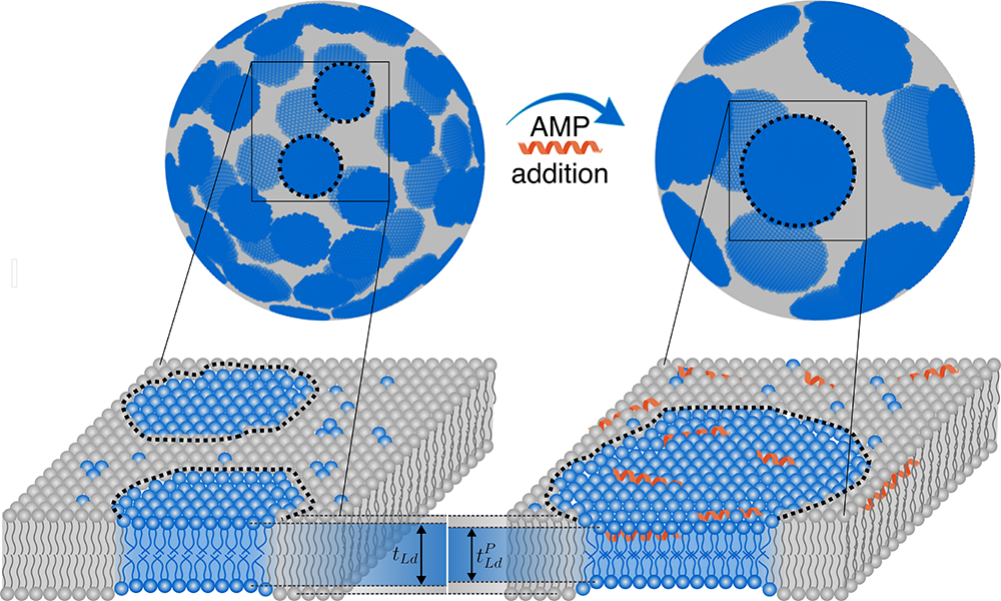
Illustration of domain growth and reduction of total boundary edge length as a result of selective peptide-induced thinning of the *L*_d_ phase.

The substantial growth of the lipid rafts upon the addition of peptides suggests a significant increase in the line tension between the two phases. This is somewhat surprising, as one might initially expect the peptides to act as “lineactants”, accumulate at the contact line between the phases, as observed by Su et al.^42^ for some of the peptides, and reduce the interfacial line tension in a similar way to their 2D analogues, surfactants.^45^ However, such a reduction of the line tension and lowering of the boundary energy would cause a reduction in the domain size. This is contrary to the observed growth of the domains and is also inconsistent with the increased *T*_m_ observed with DSC, suggesting that the peptides do not behave as lineactants in this case. The increase in energy due to higher line tension is accommodated by a reduction in the total domain edge length, achieved through domain coalescence and growth. An illustration of this process is presented in Figure 8. Both Heberle et al.^29^ and García-Sáez et al.^30^ show that increased line tension leads to increased raft size. Also, in both cases, the line tension is increased by increasing thickness mismatch between the phases by selective reduction of the thickness of the *L*_d_ phase. In the former, this is achieved by increasing the ratio of DOPC to POPC, while García-Sáez et al.^30^ uses lipids with shorter acyl chains in the unsaturated phase. Although the exact process by which the peptide increases the line tension is not directly discernible from the SANS data alone, AMPs have often been reported to affect the thickness of lipid membranes.^46−49^ Hence, an increased thickness mismatch resulting from the preferential thinning of the *L*_d_ phase, as suggested by SAXS, emerges as a possible mechanism.

## Conclusion

In this work, we investigated the effect of several natural and diverse antimicrobial peptides on the lipid raft structure, using a well characterized model system of phase separating LUVs with tunable raft size. Using small-angle neutron scattering, we show that upon exposure to the AMPs, the rafts coalesce and grow into significantly larger domains. Additionally, the bovine AMP indolicidin also seems to induce rafts in the initially non-raft-forming control. Contrast variation SANS and SAXS experiments reveal that the peptides insert into the bilayer and reduce the average bilayer thickness while simultaneously increasing the thickness heterogeneity. Further SAXS studies on the individual *L*_d_ and *L*_o_ phases and single-lipid LUVs composed entirely of DSPC, POPC, or DOPC reveal a substantial and concentration dependent thinning of the *L*_d_ phase and unsaturated DOPC and POPC bilayers upon peptide addition. The *L*_o_ phase and the saturated DSPC bilayer, on the other hand, do not display the same degree of thinning. This is supported by the DSC experiments showing stabilization of the ordered phase upon the addition of AMPs. These results suggest increased line tension as a result of the preferential thinning of the unsaturated phase, leading to the observed raft growth. The results are of vital importance in showing the multifaceted membrane interactions associated with surface-active peptides. This work provides substantial insight into the molecular effects of surface-active AMPs on model membranes, which may be relevant to real cells, e.g., related to the mode of action and the associated cytotoxicity of antimicrobial peptides. Additionally, it provides new insight into the complex mechanisms involving rafts that may have relevance to human cells and associated pathologies such as neurodegenerative diseases and cancer.

## Supporting Information Available

Detailed model descriptions of the SANS and SAXS models, additional SAXS data and analysis, additional discussion on peptide insertion, and complete model fit parameters for all scattering data (PDF)

## References

[ref1] Nichols B. Without a raft. Nature 2005, 436, 638–639. 10.1038/436638a. 16079831

[ref2] Munro S. Lipid Rafts. Cell 2003, 115, 377–388. 10.1016/S0092-8674(03)00882-1. 14622593

[ref3] Hancock J. F. Lipid rafts: contentious only from simplistic standpoints. Nat. Rev. Mol. Cell Biol. 2006, 7, 456–462. 10.1038/nrm1925. 16625153 PMC2782566

[ref4] Simons K.; Ikonen E. Functional rafts in cell membranes. Nature 1997, 387, 569–572. 10.1038/42408. 9177342

[ref5] Simons K.; Toomre D. Lipid rafts and signal transduction. Nat. Rev. Mol. Cell Biol. 2000, 1, 31–39. 10.1038/35036052. 11413487

[ref6] Sezgin E.; Levental I.; Mayor S.; Eggeling C. The mystery of membrane organization: composition, regulation and roles of lipid rafts. Nat. Rev. Mol. Cell Biol. 2017, 18, 361–374. 10.1038/nrm.2017.16. 28356571 PMC5500228

[ref7] Varshney P.; Yadav V.; Saini N. Lipid rafts in immune signalling: current progress and future perspective. Immunology 2016, 149, 13–24. 10.1111/imm.12617. 27153983 PMC4981613

[ref8] Ripa I.; Andreu S.; López-Guerrero J. A.; Bello-Morales R. Membrane Rafts: Portals for Viral Entry. Frontiers in Microbiology 2021, 12, 631274 10.3389/fmicb.2021.631274. 33613502 PMC7890030

[ref9] Rios F. J. O.; Ferracini M.; Pecenin M.; Koga M. M.; Wang Y.; Ketelhuth D. F. J.; Jancar S. Uptake of oxLDL and IL-10 Production by Macrophages Requires PAFR and CD36 Recruitment into the Same Lipid Rafts. PLoS One 2013, 8, e76893 10.1371/journal.pone.0076893. 24130805 PMC3793910

[ref10] Sorci-Thomas M. G.; Thomas M. J. Microdomains, Inflammation, and Atherosclerosis. Circ. Res. 2016, 118, 679–691. 10.1161/CIRCRESAHA.115.306246. 26892966 PMC5291489

[ref11] Greenlee J. D.; Subramanian T.; Liu K.; King M. R. Rafting Down the Metastatic Cascade: The Role of Lipid Rafts in Cancer Metastasis, Cell Death, and Clinical Outcomes. Cancer Res. 2021, 81, 5–17. 10.1158/0008-5472.CAN-20-2199. 32999001 PMC7952000

[ref12] Vanounou S.; Parola A. H.; Fishov I. Phosphatidylethanolamine and phosphatidylglycerol are segregated into different domains in bacterial membrane. A study with pyrene-labelled phospholipids. Mol. Microbiol. 2003, 49, 1067–1079. 10.1046/j.1365-2958.2003.03614.x. 12890029

[ref13] Barák I.; Muchová K. The Role of Lipid Domains in Bacterial Cell Processes. International Journal of Molecular Sciences 2013, 14, 4050–4065. 10.3390/ijms14024050. 23429192 PMC3588084

[ref14] Dupuy P.; Gutierrez C.; Neyrolles O. Modulation of bacterial membrane proteins activity by clustering into plasma membrane nanodomains. Mol. Microbiol. 2023, 120, 502 10.1111/mmi.15105. 37303242

[ref15] O’Leary E. I.; Lee J. C. Interplay between α-synuclein amyloid formation and membrane structure. Biochimica et Biophysica Acta (BBA) - Proteins and Proteomics 2019, 1867, 483–491. Lipid–protein interactions in amyloid formation 10.1016/j.bbapap.2018.09.012. 30287222 PMC6445794

[ref16] Bahar A.; Ren D. Antimicrobial Peptides. Pharmaceuticals 2013, 6, 1543–1575. 10.3390/ph6121543. 24287494 PMC3873676

[ref17] Zanetti M. Cathelicidins, multifunctional peptides of the innate immunity. Journal of Leukocyte Biology 2004, 75, 39–48. 10.1189/jlb.0403147. 12960280

[ref18] Nielsen J. E.; Bjørnestad V. A.; Pipich V.; Jenssen H.; Lund R. Beyond structural models for the mode of action: How natural antimicrobial peptides affect lipid transport. J. Colloid Interface Sci. 2021, 582, 793–802. 10.1016/j.jcis.2020.08.094. 32911421

[ref19] Sparr E.; Linse S. Lipid-protein interactions in amyloid formation. Biochimica et Biophysica Acta (BBA) - Proteins and Proteomics 2019, 1867, 455–457. 10.1016/j.bbapap.2019.03.006. 30948160

[ref20] Taylor D. R.; Hooper N. M. Role of lipid rafts in the processing of the pathogenic prion and Alzheimers amyloid-β proteins. Seminars in Cell & Developmental Biology 2007, 18, 638–648. 10.1016/j.semcdb.2007.07.008. 17822928

[ref21] Azouz M.; Cullin C.; Lecomte S.; Lafleur M. Membrane domain modulation of Aβ_1–42_ oligomer interactions with supported lipid bilayers: an atomic force microscopy investigation. Nanoscale 2019, 11, 20857–20867. 10.1039/C9NR06361G. 31657431

[ref22] McHenry A. J.; Sciacca M. F.; Brender J. R.; Ramamoorthy A. Does cholesterol suppress the antimicrobial peptide induced disruption of lipid raft containing membranes?. Biochimica et Biophysica Acta (BBA) - Biomembranes 2012, 1818, 3019–3024. 10.1016/j.bbamem.2012.07.021. 22885355 PMC3455134

[ref23] Pokorny A.; Almeida P. F. F. Permeabilization of Raft-Containing Lipid Vesicles by δ-Lysin: A Mechanism for Cell Sensitivity to Cytotoxic Peptides. Biochemistry 2005, 44, 9538–9544. 10.1021/bi0506371. 15996108

[ref24] Lingwood D.; Simons K. Lipid Rafts As a Membrane-Organizing Principle. Science 2010, 327, 46–50. 10.1126/science.1174621. 20044567

[ref25] Veatch S. L.; Keller S. L. Separation of Liquid Phases in Giant Vesicles of Ternary Mixtures of Phospholipids and Cholesterol. Biophys. J. 2003, 85, 3074–3083. 10.1016/S0006-3495(03)74726-2. 14581208 PMC1303584

[ref26] Veatch S. L.; Keller S. L. Miscibility Phase Diagrams of Giant Vesicles Containing Sphingomyelin. Phys. Rev. Lett. 2005, 94, 148101 10.1103/PhysRevLett.94.148101. 15904115

[ref27] Zhao J.; Wu J.; Heberle F. A.; Mills T. T.; Klawitter P.; Huang G.; Costanza G.; Feigenson G. W. Phase studies of model biomembranes: Complex behavior of DSPC/DOPC/Cholesterol. Biochimica et Biophysica Acta (BBA) - Biomembranes 2007, 1768, 2764–2776. 10.1016/j.bbamem.2007.07.008. 17825247 PMC2701629

[ref28] Heberle F. A.; Wu J.; Goh S. L.; Petruzielo R. S.; Feigenson G. W. Comparison of Three Ternary Lipid Bilayer Mixtures: FRET and ESR Reveal Nanodomains. Biophys. J. 2010, 99, 3309–3318. 10.1016/j.bpj.2010.09.064. 21081079 PMC2980711

[ref29] Heberle F. A.; Petruzielo R. S.; Pan J.; Drazba P.; Kučerka N.; Standaert R. F.; Feigenson G. W.; Katsaras J. Bilayer Thickness Mismatch Controls Domain Size in Model Membranes. J. Am. Chem. Soc. 2013, 135, 6853–6859. 10.1021/ja3113615. 23391155

[ref30] García-Sáez A. J.; Chiantia S.; Schwille P. Effect of Line Tension on the Lateral Organization of Lipid Membranes. J. Biol. Chem. 2007, 282, 33537–33544. 10.1074/jbc.M706162200. 17848582

[ref31] Ouweneel A. B.; Thomas M. J.; Sorci-Thomas M. G. The ins and outs of lipid rafts: functions in intracellular cholesterol homeostasis, microparticles, and cell membranes. J. Lipid Res. 2020, 61, 676–686. 10.1194/jlr.TR119000383. 33715815 PMC7193959

[ref32] Nickels J. D.; Cheng X.; Mostofian B.; Stanley C.; Lindner B.; Heberle F. A.; Perticaroli S.; Feygenson M.; Egami T.; Standaert R. F.; Smith J. C.; Myles D. A. A.; Ohl M.; Katsaras J. Mechanical Properties of Nanoscopic Lipid Domains. J. Am. Chem. Soc. 2015, 137, 15772–15780. 10.1021/jacs.5b08894. 26415030

[ref33] Krzyzanowski N.; Porcar L.; Perez-Salas U. A Small-Angle Neutron Scattering, Calorimetry and Densitometry Study to Detect Phase Boundaries and Nanoscale Domain Structure in a Binary Lipid Mixture. Membranes 2023, 13, 323 10.3390/membranes13030323. 36984710 PMC10051979

[ref34] Konyakhina T. M.; Wu J.; Mastroianni J. D.; Heberle F. A.; Feigenson G. W. Phase diagram of a 4-component lipid mixture: DSPC/DOPC/POPC/chol. *Biochimica et Biophysica Acta (BBA) -*. Biomembranes 2013, 1828, 2204–2214. 10.1016/j.bbamem.2013.05.020. PMC3738200 23747294

[ref35] Nielsen J. E.; Lind T. K.; Lone A.; Gerelli Y.; Hansen P. R.; Jenssen H.; Cárdenas M.; Lund R. A biophysical study of the interactions between the antimicrobial peptide indolicidin and lipid model systems. Biochimica et Biophysica Acta (BBA) - Biomembranes 2019, 1861, 1355–1364. 10.1016/j.bbamem.2019.04.003. 30978313

[ref36] Nielsen J. E.; Bjørnestad V. A.; Lund R. Resolving the structural interactions between antimicrobial peptides and lipid membranes using small-angle scattering methods: the case of indolicidin. Soft Matter 2018, 14, 8750–8763. 10.1039/C8SM01888J. 30358793

[ref37] Nielsen J. E.; Prévost S. F.; Jenssen H.; Lund R. Impact of antimicrobial peptides on E. coli-mimicking lipid model membranes: correlating structural and dynamic effects using scattering methods. Faraday Discuss. 2021, 232, 203–217. 10.1039/D0FD00046A. 34590103

[ref38] Kučerka N.; Pencer J.; Sachs J. N.; Nagle J. F.; Katsaras J. Curvature Effect on the Structure of Phospholipid Bilayers. Langmuir 2007, 23 (3), 1292–1299. 10.1021/la062455t. 17241048 PMC2720570

[ref39] Tully M. D.; et al. BioSAXS at European Synchrotron Radiation Facility– Extremely Brilliant Source: BM29 with an upgraded source, detector, robot, sample environment, data collection and analysis software. Journal of Synchrotron Radiation 2023, 30, 258–266. 10.1107/S1600577522011286. 36601945 PMC9814054

[ref40] Narayanan T.; Sztucki M.; Zinn T.; Kieffer J.; Homs-Puron A.; Gorini J.; Van Vaerenbergh P.; Boesecke P. Performance of the time-resolved ultra-small-angle X-ray scattering beamline with the Extremely Brilliant Source. J. Appl. Crystallogr. 2022, 55, 98–111. 10.1107/S1600576721012693. 35145357 PMC8805168

[ref41] Nielsen J. E.; Koynarev V. R.; Lund R. Peptide meets membrane: Investigating peptide-lipid interactions using small-angle scattering techniques. Curr. Opin. Colloid Interface Sci. 2023, 66, 101709 10.1016/j.cocis.2023.101709.

[ref42] Su J.; Marrink S. J.; Melo M. N. Localization Preference of Antimicrobial Peptides on Liquid-Disordered Membrane Domains. Frontiers in Cell and Developmental Biology 2020, 8, 350 10.3389/fcell.2020.00350. 32509780 PMC7248343

[ref43] Chappa V.; Smirnova Y.; Komorowski K.; Müller M.; Salditt T. The effect of polydispersity, shape fluctuations and curvature on small unilamellar vesicle small-angle X-ray scattering curves. J. Appl. Crystallogr. 2021, 54, 557–568. 10.1107/S1600576721001461. 33953656 PMC8056763

[ref44] Eicher B.; Heberle F. A.; Marquardt D.; Rechberger G. N.; Katsaras J.; Pabst G. Joint small-angle X-ray and neutron scattering data analysis of asymmetric lipid vesicles. J. Appl. Crystallogr. 2017, 50, 419–429. 10.1107/S1600576717000656. 28381971 PMC5377341

[ref45] Trabelsi S.; Zhang S.; Lee T. R.; Schwartz D. K. Linactants: Surfactant Analogues in Two Dimensions. Phys. Rev. Lett. 2008, 100, 037802 10.1103/PhysRevLett.100.037802. 18233038

[ref46] Huang H. W. Molecular mechanism of antimicrobial peptides: The origin of cooperativity. *Biochimica et Biophysica Acta (BBA) -*. Biomembranes 2006, 1758, 1292–1302. 10.1016/j.bbamem.2006.02.001. 16542637

[ref47] Sevcsik E.; Pabst G.; Richter W.; Danner S.; Amenitsch H.; Lohner K. Interaction of LL-37 with Model Membrane Systems of Different Complexity: Influence of the Lipid Matrix. Biophys. J. 2008, 94, 4688–4699. 10.1529/biophysj.107.123620. 18326643 PMC2397346

[ref48] Pabst G.; Grage S. L.; Danner-Pongratz S.; Jing W.; Ulrich A. S.; Watts A.; Lohner K.; Hickel A. Membrane Thickening by the Antimicrobial Peptide PGLa. Biophys. J. 2008, 95, 5779–5788. 10.1529/biophysj.108.141630. 18835902 PMC2599817

[ref49] Grage S. L.; Afonin S.; Kara S.; Buth G.; Ulrich A. S. Membrane Thinning and Thickening Induced by Membrane-Active Amphipathic Peptides. Frontiers in Cell and Developmental Biology 2016, 4, 65 10.3389/fcell.2016.00065. 27595096 PMC4999517

