# Supplementary material for: Antimicrobial Peptides Increase Line Tension in Raft-Forming Lipid Membranes
Source: J Am Chem Soc. 2024 Jul 17;146(30):20891–903. doi: 10.1021/jacs.4c05377 (PMC11295182; doi:10.1021/jacs.4c05377)
Supplement: Supplementary file 1 — ja4c05377_si_001.pdf [file ja4c05377_si_001.pdf]

Supporting Information:

Antimicrobial Peptides Increase Line Tension in Raft-forming Lipid Membranes

Vladimir Rosenov Koynarev,[†] Kari Kristine Almåsvold Borgos,[†] Joachim Kohlbrecher,[‡] Lionel Porcar,[¶] Josefine Eilsø Nielsen,[†] and Reidar Lund[*,†,§]

†*Department of Chemistry, University of Oslo, Postboks 1033 Blindern, 0315 Oslo, Norway*

‡*Laboratory for Neutron Scattering and Imaging, Paul Scherrer Institut, Villigen 5232, Switzerland*

¶*Institut Laue-Langevin, 71 Av. des Martyrs, 38000 Grenoble, France.*

§*Hylleraas Centre for Quantum Molecular Sciences, University of Oslo,Postboks 1033 Blindern, 0315 Oslo, Norway*

E-mail: reidar.lund@kjemi.uio.no

S1. Modeling SANS scattering from the lipid rafts

S1.1. The Debye equation

In order to describe the lateral contrast between the domains and the continuous phase, liposomes are modeled by distributing a large number of small solid spherical beads on the surface of a sphere and labeling some of the points as belonging to the domains and the rest as belonging to the continuous phase (Fig. S1), in accordance with the area fraction of each phase. Notably the size of the spherical shell corresponds to the size of the liposome, while the size of the beads are much smaller and not individually resolvable within the experimental Q range. The Debye equation can then be used to describe the scattering from this complex

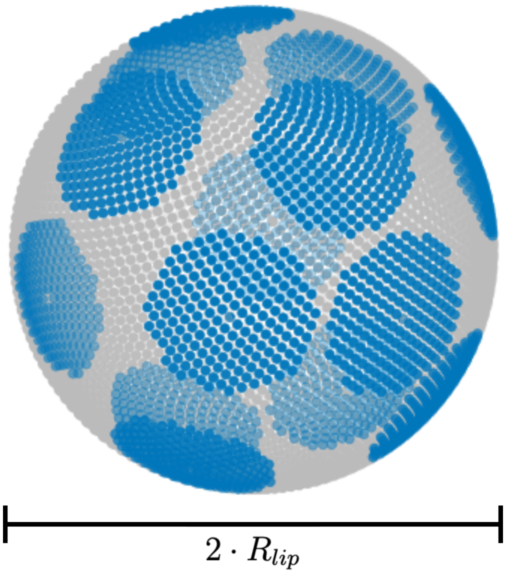

Figure S1: Example of the geometry used to model the SANS scattering from raft forming liposomes. $N = 6000$ spherical beads with radius r_b are distributed on a surface of a sphere with radius R_{lip}. In this particular case, $M = 3467$ are labeled as continuous phase beads (gray), while $K = 2533$ are labeled as domain beads (blue), grouped in 14 domains randomly distributed on the sphere surface with a small excluded area between the domains.

object made up of many simple beads. The scattering amplitude of each individual spherical bead, with radius r_b is well known and given as a function of the scattering vector Q as:

$$A(Q, r_b) = \frac{3\left(\sin(Qr_b) - Qr_b \cos(Qr_b)\right)}{(Qr_b)^3}. \tag{S1}$$

For an arbitrary object comprised of N such solid beads the intensity is then given by the Debye equation as,

$$I_{Debye}(Q) = \frac{1}{V_{tot}} \sum_{i}^{N} \sum_{j}^{N} v_i v_j A(Q)_i A(Q)_j \Delta\rho_i \Delta\rho_j \frac{\sin(Qr_{ij})}{Qr_{ij}}, \tag{S2}$$

where $A_i(Q)$ and $A_j(Q)$ are the are the spherical scattering amplitudes of sphere i and j respectively, while v_i and v_j are the volumes and $\Delta\rho_i$ and $\Delta\rho_j$ are the scattering length densities relative to the solvent ($\Delta\rho_i = \rho_i - \rho_{sol}$) for the respective spheres. Lastly, r_{ij} is the euclidean distance between the center of the two spheres, and the scattering is normalized by the total volume of scatters V_{tot}.

The domain forming liposomes are modeled by distributing these N spherical beads of radius $r_b = 5$ Å uniformly, and approximately regularly, on a spherical surface with radius R_{lip}, where $r_b \ll R_{lip}$, forming what is known as a *Fibonacci sphere*. From these N beads, N_{dom} are chosen to be the center of domains, and the neighboring beads within a half-angle, α_{dom}, of each domain center are labeled as domain beads (blue beads in Fig. S1), while the remaining beads are labeled as continuous phase (gray beads in Fig. S1). This results in

a total of K beads belonging to domains and M beads belonging to the continuous phase, with $K + M = N$. Both populations of beads have the same radius, and therefore the same volume, v_0, and only differ in the relative scattering length densities. Using this and keeping in mind that $V_{tot} = Nv_0$ the Debye equation can be expressed as:

$$I_{Debye}(Q) = \frac{v_0^2 A(Q, r_b)^2}{N v_0} \left[\sum_i^N \sum_j^N \Delta\rho_i \Delta\rho_j \frac{\sin(Qr_{ij})}{Qr_{ij}} + N \right], \tag{S3}$$

The cases where $i = j$ result in $r_{ij} = 0$. These are explicitly handled by using $\lim_{x\to 0} [\sin(x)/x] = 1$, which sums up to N. The Debye equation can then be split up into separate terms for the domain and continuous phase beads, and the cross-terms between the two:

$$\begin{aligned} I_{Debye}(Q) = \frac{v_0 A(Q, r_b)^2}{N} & \left[\Delta\rho_d^2 \left(\sum_i^K \sum_j^K \frac{\sin(Qr_{ij})}{Qr_{ij}} + K \right) + \right. \\ & \left. \Delta\rho_c^2 \left(\sum_i^M \sum_j^M \frac{\sin(Qr_{ij})}{Qr_{ij}} + M \right) + 2\Delta\rho_d \Delta\rho_c \sum_i^K \sum_j^M \frac{\sin(Qr_{ij})}{Qr_{ij}} \right], \end{aligned} \tag{S4}$$

where $\Delta\rho_d$ and $\Delta\rho_c$ respectively, are the SLDs for the domain and continuous phase beads relative to the buffer. As a suitable discretization of the scattering intensity using the Debye equation requires a large value for N, equation (S4) becomes computationally costly. Following the approach presented in,[S1] the computational cost can be reduced by computing a histogram, $h(r_k)$, of the pairwise distances between the beads for each of the three terms inside the square brackets, resulting in the final version of the Debye equation:

$$\begin{aligned} I_{Debye}(Q) = \frac{v_0 A(Q, r_b)^2}{N} & \left[\Delta\rho_d^2 \left(\sum_k^{N_h} h_d(r_k) \frac{\sin(Qr_k)}{Qr_k} + K \right) + \right. \\ & \left. \Delta\rho_c^2 \left(\sum_k^{N_h} h_c(r_k) \frac{\sin(Qr_k)}{Qr_k} + M \right) + 2\Delta\rho_d \Delta\rho_c \sum_k^{N_h} h_{dc}(r_k) \frac{\sin(Qr_k)}{Qr_k} \right]. \end{aligned} \tag{S5}$$

Here h_d and h_c are the histograms of pairwise distances for the domain and continuous phase beads respectively, while h_{dc} is the histogram of distances *between* the two populations. N_h is the number of bins in the histograms and r_k is the distance of the kth bin. With this approach, quick and well optimized algorithms can be used to compute the pairwise distances, while the more costly sinus terms need only to be computed $3N_h$ times, compared to the $(N^2 - N)$ computations initially required. This achieves a significant reduction in computational cost as $N_h \ll N$. In present case each liposomes was divided into $N = 6000$ beads.

S1.2. Distributing the domains on the liposome surface

The combined area covered by domains, as a fraction of the total surface area of the liposome, is given by A_f. Dividing this total area by the number of domains N_{dom} gives the fraction of surface area covered by a single domain, $A_{f,dom} = A_f/N_{dom}$, in the form of a spherical cap. The domain half angle, α_{dom} defined as the angle between the vector going from the center of the liposome to the center of the domain, and the vector going from the center of the liposome to the edge of the domain, is then given by $\alpha_{dom} = \cos^{-1}(1 - 2A_{f,dom})$.

Having determined the domain half angle, N_{dom} beads are selected as center-points for the domains *at random*, but with the restriction that the angle between any two domain centers (angle between the two vectors going from the center of the liposome to each of the two domain centers) has to be equal or greater than $2 \cdot f_{ex} \cdot \alpha_{dom}$, where $f_{ex} \approx 1.03$ was used. This ensures that the domains are not overlapping, nor touching with a small excluded area in between them. This small excluded surface area, corresponding to roughly 3 % larger domains resulted in a better fit than using $f_{ex} = 1$, i.e. no excluded surface and potentially touching domains. While case of having the maximum possible separation of the domains resulted in significantly worse fits.

S1.3. Accounting for instrumental smearing and size polydispersity

Following the construction of the liposome and definition of the domain and continuous phase points, the scattering intensity can be computed using equation (S5), however as this is SANS data obtained using a relative broad range of neutron wavelengths ($\Delta\lambda/\lambda = 0.1$), a lenient collimation of the beam and a finite detector resolution, the instrumental resolution leads to significant smearing of the data. These effects are taken into account through the convolution of computed intensity and instrumental resolution function as described by Pedersen.[S2,S3] Briefly, the convoluted intensity is numerically computed for each nominal (experimental) Q value, Q_i, as:

$$I_{res}(Q_i) = \sum_{j=1}^{N_q} R(Q_i, q_j) I_{Debye}(q_j) \Delta q \tag{S6}$$

where $R(Q, q)$ is the resolution function describing the distribution of scattering vectors q contributing to the nominal scattering vector Q, given as:

$$R(Q, q) = \frac{q}{\sigma^2} \exp\left(-\frac{1}{2}\frac{(q^2 + Q^2)}{\sigma^2}\right) I_0, \tag{S7}$$

where I_0 is the fist kind, 0 order Bessel function, and the variance σ^2 is given by

$$\sigma^2 = Q^2\sigma_\lambda^2 + \left(\frac{4\pi}{\lambda}\right)^2 \sigma_\theta^2 \tag{S8}$$

here $\sigma_\lambda = \Delta\lambda(2\sqrt{2\ln(2)})$, is the deviation due to wavelength distribution, while σ_θ is the deviation due to finite collimation and detector resolution and is determined by fitting a Gaussian to the measurement of the empty beam. Ultimately, for each nominal Q_i value, $N_q = 21$, equidistant q_j values are taken in the interval $[Q_i - 3Q_i\sigma,\ Q_i + 3Q_i\sigma]$. The intensity

at each $q_{i,j}$ value is computed using the Debye equation (S5) and the smeared is intensity evaluated using eq. (S6).

In addition to the experimental resolution, the polydispersity in the liposome sizes also leads to smearing of the scattering intensity and has to be considered. Assuming that the radius of the liposomes follows a Gaussian distribution centered on the average radius, R_{lip}, with a standard deviation of σ_R, N_R values for the radius R_k are chosen equidistantly in the interval from $[R_{lip} - 3\sigma_R R_{lip},\ R_{lip} + 3\sigma_R R_{lip}]$. For each of these R_k radii a liposome with domains is generated with the aforementioned method and the resolution convoluted intensity $I(Q, R_k)$ is computed as described. An weighted average of these intensities is computed, where the weights, w_k, correspond to the normal distribution probability of the appropriate radius, resulting in.

$$I_{avr}(Q) = \frac{\sum_{k=1}^{N_R} I_{res}(Q, R_k) \cdot w_k}{\sum_{k=1}^{N_R} w_k} \tag{S9}$$

Lastly the intensity is scaled with an constant C to account for experimental parameters such as concentration, sample path length, detector distance etc. The final intensity is given as:

$$I(Q) = C \cdot I_{avr}(Q). \tag{S10}$$

In the fitting procedure, the values reported in[S4] are used for A_f, $\Delta\rho_d$ and $\Delta\rho_c$, and only allowed to vary slightly around the reported values. While R_{lip}, σ_R, f_{ex} and N_{dom} are the main free fitting parameters that are varied, but kept within physically meaningful values. The following parameters were chosen to allow for appropriate discretization while keeping the computational cost reasonable, and kept fixed to the presented values across all fits: $N = 6000$, $N_h = 200$, $N_q = 21$ and $N_R = 101$.

S1.4. Preferential insertion of peptide

If Indolicidin were to insert preferentially into one of the two phases in the raft forming vesicles, the contrast and resulting scattering intensity might change due to two effects. Firstly as the peptide has a different neutron scattering length density (SLD) to both that of the lipid heads and that of the lipid tails in the two phases, insertion of the peptide into one phase will change the SLD of that phase. Secondly, as the peptide is added in a non-negligible 1:20 PL molar ratio, and due to the close to double volume of Indolicidin ($V_p \approx 2341$ Å^3) compared to the average volume of a single phospholipid ($V_L \approx 1225$ Å^3), it is reasonable that the area fraction of the phase that the peptide is inserted into will increase to accommodate the extra volume. Both of these factors can conceivably affect the scattering intensity and should be considered as possible explanation of the observed increase in scattering intensity.

To thoroughly explore this possibility, we took the optimized model parameters for the large raft forming vesicles *without* peptide, and subsequently, assumed that all of the peptide inserted into one of the phases and the SLD of that phase changed accordingly using equation (S11). This was done for both complete peptide insertion in the L_d and L_o respectively, but initially the area fraction of the respective phases was not changed. All other parameters,

including the number of domains, were kept fixed. The resulting scattering curves are shown with solid lines in figure S2. The scattering curves resulting from a change in *both* SLD and area fraction of the peptide to accommodate for the peptide volume are shown with dotted lines.

It is clear that even if the peptide inserts completely into one of the phases the resulting change in contrast and area fraction of that phase is insufficient to describe the increase in scattering intensity observed upon peptide addition (Fig 2.B).

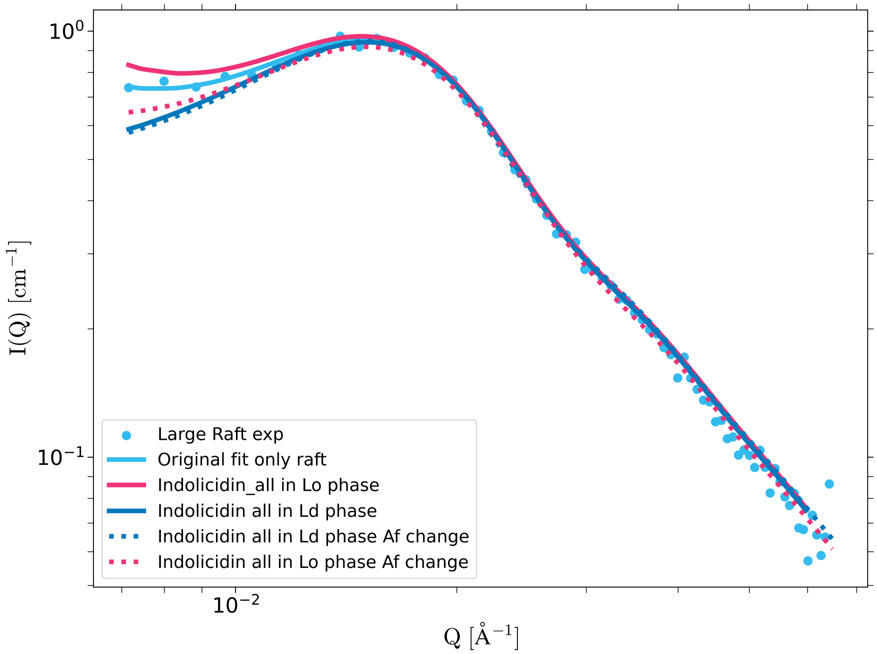

Figure S2: Experimental SANS data (scattered circles) of large raft forming vesicles with the original fit (solid light blue line). Completely discriminatory peptide insertion in Ld or L_o phases, but with no resulting change in phase area fraction are shown with solid dark blue and magenta lines respectively. Complete discriminatory insertion and a corresponding increase in phase area fraction are shown with dotted dark blue and magenta lines for the L_d and L_o phases respectively.

The scattering length density of the given phase with inserted peptide $\rho_{phase}^{Peptide}$ was calculated as:

$$\rho_{phase}^{Peptide} = \frac{\chi_p^{phase} V_p \rho_p + (1 - \chi_p^{phase}) V_L \rho_{phase}}{\chi_p^{phase} V_p + (1 - \chi_p^{phase}) V_L}, \tag{S11}$$

where χ_p^{phase} is the molar fraction of peptide in the phase if *all* peptide was to insert there, calculated from the global peptide molar fraction and the molar fraction of the phase as reported in SI of ref,[S4] with $\chi_p^{L_d} = 0.143$ and $\chi_p^{L_o} = 0.077$. $\rho_p = 0.241$ fm/Å^3 is the SLD

of the peptide with all liable H exchanged with D, while ρ_{phase} is the SLD of the given phase as in the original model fit with no peptide ($\rho_{L_d} = 0.027$ fm/Å^3 and $\rho_{L_o} = 0.275$ fm/Å^3). V_p and V_L are the molecular volumes of the peptide and average lipid respectively.

S1.5. Lipid rafts with added peptide

The SANS scattering from the pure liposomes (i.e. no added peptide) can be explained well using a single, well defined number of domains for each composition. This is not the case when peptide is added to the liposomes that form lipid rafts. In order to fit the data adequately a combination of the scattering from liposomes with multiple large rafts and single very large raft, similar to a Janus particle, is necessary. In figure S3 the SANS data from Medium raft with added peptide is shown with the fit from a single domain (dashed line), as well as with 14 domains (dotted line), with the corresponding geometrical models. In both cases the total area fraction of domains is the same, and the data is adequately fitted when the combined scattering from the two is used (solid blue line). This is very similar to the strategy used to explain the scattering from the D7 composition which was reported to from very large rafts by Heberle *et al*,[S4] where the intensity was modeled using a combination of $N(raft) = 1$ raft (Janus particle) and $N(raft) = 4$ rafts.

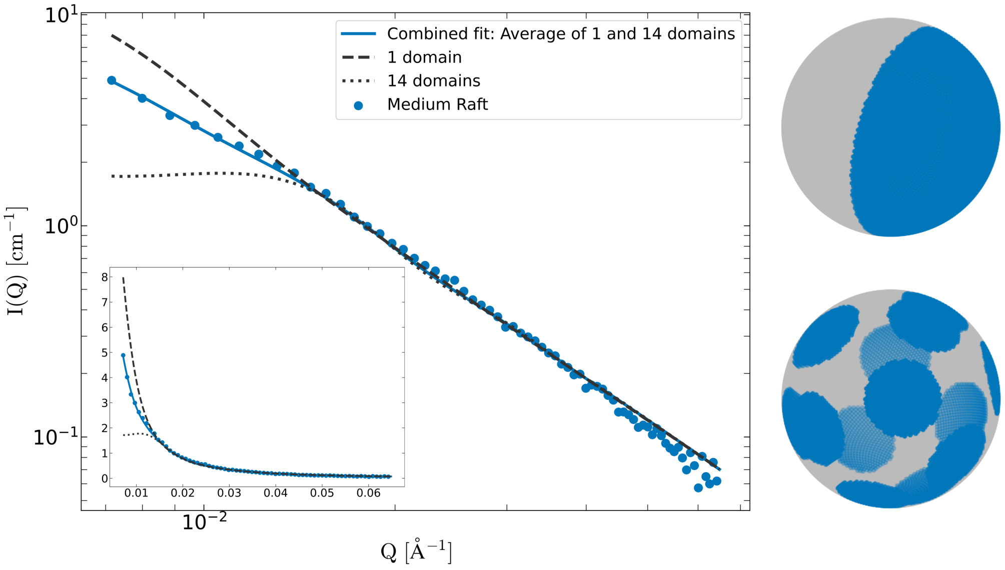

Figure S3: SANS data of the Medium raft composition with Indolicidin added in a 1:20 peptide:lipid molar ratio, fitted using only a single domain (dashed line), or only 14 domains (dotted line) and the combination of the two (solid line). The geometrical models are shown to the right, where the same total area fraction is divided into 1 (top) and 14 domains (bottom) domains, respectively.

Such a combined model was used to fit the data for the Large, Medium and Small raft composition *with* added peptide, where the combined scattering from $N_{dom} = 1$ and respectively $N_{dom} = 13$, $N_{dom} = 14$ and $N_{dom} = 15$, was used.

S1.6 SANS model fit parameters

The optimized model parameters resulting from the fit analysis using the discrete model to analyse the data from the raft-forming vesicles are shown in the tables below. Table S1 corresponds to the data presented in figure 2 of the main paper, while table S2 corresponds to figure 8. In the tables below, N_{dom} is the number of domains that the L_d phase is divided into, and $A_{f,Ld}$ is the total area fraction of the L_d phase. N_{dom} is a free fitting parameter that can only take integer values, while the area fraction is taken from phase composition reported by Heberle *et al*[S4] and only allowed to vary slightly. This is also the case for ρ_{Ld} and ρ_{Lo} which are the scattering length densities of the L_d and L_o phases respectively, while the buffer SLD ρ_s is calculated and kept constant. From the remaining fitting parameters, R and σ_R are the average and width of the Gaussian vesicle radius distribution, respectively. Lastly, in case of the vesicles with added peptide, f_{janus} is the fraction of Janus particles where the entire L_d phase is in a single domain, as discussed above.

Table S1: SANS model fit parameters, with estimated uncertainty for raft forming vesicles without peptide, and with Indolicidin added in a 1:20 PL ratio. *For the No raft vesicle without peptide a homogeneous model was used where the individual beads are homogeneously distributed to describe the flat scattering observed, however due to the general lack of any features in the SANS curve uncertainty estimates could not be reliably determined.

**Parameter**	**L**	**M**	**S**	**No raft***	**L 1:20**	**M 1:20**	**S 1:20**	**No raft 1:20**
N_{dom}	26 ± 1	50 ± 2	56 ± 2	n.a	13 ± 1	14 ± 1	15 ± 1	250 ± 25
$A_{f,Ld}$	0.43 ± 0.02	0.42 ± 0.02	0.31 ± 0.015	0.43	0.43 ± 0.02	0.42 ± 0.02	0.31 ± 0.015	0.2 ± 0.05
R [Å]	630 ± 25	600 ± 20	680 ± 23	590	650 ± 22	600 ± 24	680 ± 29	600 ± 20
σ_R	0.12 ± 0.03	0.07 ± 0.02	0.028 ± 0.08	0.02	0.12 ± 0.034	0.07 ± 0.02	0.25 ± 0.07	0.39 ± 0.11
ρ_s [fm/Å^3]	0.185	0.185	0.185	0.185	0.185	0.185	0.185	0.185
ρ_{Ld} [fm/Å^3]	0.027 ± 0.003	0.024 ± 0.003	0.048 ± 0.006	0.027	0.027 ± 0.003	0.024 ± 0.003	0.048 ± 0.006	0.027 ± 0.003
ρ_{Lo} [fm/Å^3]	0.275 ± 0.005	0.265 ± 0.005	0.275 ± 0.005	0.275	0.275 ± 0.005	0.265 ± 0.005	0.275 ± 0.005	0.275 ± 0.005
f_{janus}	n.a.	n.a.	n.a.	n.a.	0.65 ± 0.05	0.50 ± 0.04	0.50 ± 0.04	0

Table S2: SANS model fit parameters, with estimated uncertainty for large raft forming vesicles with addition of different peptides.

**Parameter**	**L**	**L LL37 1:100**	**L MagII 1:20**	**L Aur2.2 1:20**
N_{dom}	29 ± 2	22 ± 2	20 ± 1	12 ± 1
$A_{f,Ld}$	0.44 ± 0.02	0.44 ± 0.02	0.44 ± 0.02	0.44 ± 0.02
R [Å]	600 ± 20	680 ± 23	680 ± 23	650 ± 23
σ_R	0.24 ± 0.07	0.24 ± 0.07	0.24 ± 0.07	0.24 ± 0.07
ρ_s [fm/Å^3]	0.185	0.185	0.185	0.185
ρ_{Ld} [fm/Å^3]	0.027 ± 0.003	0.027 ± 0.003	0.027 ± 0.003	0.027 ± 0.003
ρ_{Lo} [fm/Å^3]	0.275 ± 0.005	0.275 ± 0.005	0.275 ± 0.005	0.275 ± 0.005
f_{janus}	n.a.	0.4 ± 0.03	0.4 ± 0.03	0.65 ± 0.05

S2. The SAXS model

The scattering intensity from a concentric shells model with a polydisperse internal liposome radius, R_i, can be expressed by

$$I_{lip}(Q) = n_{lip} \int_0^\infty G(R_i, \sigma_{RiPD}) \cdot A_{CS}(Q, R_i)^2 dR_i, \tag{S12}$$

where, n_{lip} is the number density of the liposomes in the sample, and the integral is the convolution between a distribution function G (typically a Gaussian) and the square of the concentric shells amplitude A_{CS}, which can be expressed as:

$$A_{CS}(Q, R_i) = \sum_{j=1}^{N_S=3} \Delta\rho_j \cdot V_j \cdot A_{shell}(Q, R_j, R_{j-1}), \tag{S13}$$

where the sum goes over the $N_S = 3$ concentric spherical shells, each with with amplitude $A_{shell}(Q, R_j, R_{j-1})$ that is a function of the inner and outer radii R_{j-1} and R_j of shell j respectively. It is emphasized that in the implementation the outer radius R_j is defined by adding the thickness of the current shell t_j as well as the thicknesses of the previous shells $t_{j-1,j-2...}$ to the inner liposome radius R_i. The shell amplitude A_{shell} can be expressed as.

$$\begin{aligned} A_{shell}(Q) = \frac{1}{V_j} [&V_s(R_j) \cdot A(Q, R_j) \cdot DW(Q, \sigma_j) - \\ &V_s(R_{j-1}) \cdot A(Q, R_{j-1}) \cdot DW(Q, \sigma_{j-1})] , \end{aligned} \tag{S14}$$

where $A(Q)$ is the amplitude of a solid sphere as described in equation (S1), and $DW(Q, \sigma_j) = \exp(-Q^2\sigma_j^2/2)$ is the Debye-Waller factor and accounts for diffuse borders between the different shells. $V_s(R_j)$ is the volume of a sphere with radius R_j, V_j is the volume of shell j, and the contrast $\Delta\rho_j = \rho_j - \rho_s$ is the difference between the SLD of shell j and the SLD of the solvent, ρ_j and ρ_s respectively. The SLD of each shell is determined by its composition. Defining P_{lip} as the lipid aggregation number, i.e number of lipids per liposome, the number of cholesterol and peptide molecules per liposome can be expressed as $P_{Chol} = P_{lip} \cdot r_{ChL}$ and $P_{pep} = P_{lip} \cdot r_{PL} \cdot \chi_P$, respectively. $r_{ChL} = \frac{n_{Chol}}{n_{lip}}$ and $r_{PL} = \frac{n_{pep}}{n_{lip}}$ are the stoichiometric ratios between cholesterol and lipids, and peptide and lipids, respectively. $\chi_P = 1 - f_{P,free}$ and $f_{P,free}$ are the fractions of bound and free peptide respectively, as the peptide does not necessarily have to be completely bound to the liposome. An additional consideration concerning the peptide is that the peptide can be distributed differently in the different shells, hence the amount of peptide in shell j can be expressed as $P_{pep} \cdot f_j^P$, where f_j^P is the fraction of peptide in that shell.

The lipid aggregation number, on which the peptide and cholesterol aggregation numbers depend, can be determined using the assumption that the central hydrocarbon shell can have no voids and that no aqueous solvent is present. It is further assumed that cholesterol is in its canonical upright position such that the OH-group is in the lipid head group shell while the rest of the cholesterol molecule, labeled as $Chol_{tail}$, is in the hydrocarbon shell. Under these assumptions the volume of the hydrocarbon shell, which is given from the outer shell

radius R_2 and shell thickness t_{HC} as $V_{HC} = \frac{4\pi}{3}\left(R_2^3 - (R_2 - t_{HC})^3\right)$, has to be completely filled with lipid tails, cholesterol tails and/or peptide. Hence;

$$P_{lip} \cdot V_{tail}^{lip} + P_{lip} r_{ChL} \cdot V_{tail}^{Chol} + P_{lip} r_{PL} \chi_P f_{HC}^P \cdot V_{pep} = \frac{4\pi}{3}\left(R_2^3 - (R_2 - t_{HC})^3\right), \tag{S15}$$

and solving for P_{lip} results in:

$$P_{lip} = \frac{4\pi[R_2^3 - (R_2 - t_{HC})^3]}{3(V_{tail}^{lip} + r_{ChL} \cdot V_{tail}^{Chol} + r_{PL}\chi_P f_{HC}^P \cdot V_{pep})}, \tag{S16}$$

where V_{tail}^{lip}, V_{tail}^{Chol} and V_{pep} are the lipid tail, cholesterol tail and peptide volumes respectively, and f_{HC} is the fraction of peptide in the hydrocarbon shell. Due to the curvature of the bilayer in the spherical liposome, the inner bilayer leaflet has less volume compared to the outer leaflet, the number of lipids in the each leaflet is therefore given as $P_{lip} \cdot \chi_{leaf}^L$ with $\chi_{leaf}^L = \chi_{outer}^L = (R_2^3 - (R_2 - t_{HC}/2)^3)/(R_2^3 - (R_2 - t_{HC})^3)$ for the outer leaflet and $\chi_{inner}^L = 1 - \chi_{outer}^L$ for the inner leaflet. The volume fraction of the lipid head or tail, ϕ_j^L, cholesterol head or tail, ϕ_j^{Chol}, peptide, ϕ_j^P and solvent, ϕ_j^s in shell j are then given as:

$$\begin{aligned}
\phi_j^L &= \left(P_{lip} \cdot \chi_{leaf}^L \cdot V_j^{lip}\right)/V_j \\
\phi_j^{Chol} &= \left(P_{lip} \cdot \chi_{leaf}^L \cdot r_{ChL} \cdot V_j^{Chol}\right)/V_j \\
\phi_j^P &= \left(P_{lip} \cdot \chi_{leaf}^L \cdot r_{PL} \cdot \chi^P \cdot f_j^P \cdot V_{pep}\right)/V_j \\
\phi_j^S &= 1 - \phi_j^{lip} - \phi_j^{Chol} - \phi_j^P
\end{aligned} \tag{S17}$$

where V_j^{lip} and V_j^{Chol} is the volume of the lipid head or tail, and cholesterol head or rail fragment, depending what is appropriate for the given shell j, while V_j is the volume of that shell. Note that for the central HC (j=2) shell there is no solvent and $\phi_j^S = 0$ per definition. Finally, the scattering lengt density for shell j is given as:

$$\rho_j = \phi_j^L \rho_j^L + \phi_j^{Chol} \rho_j^{Chol} + \phi_j^P \rho_P + \phi_j^S \rho_S, \tag{S18}$$

where ρ_j^L, ρ_j^{Chol}, ρ_P and ρ_S are the SLDs of the lipid fragment, cholesterol fragment, peptide and solvent respectively.

Lastly, a small fraction of 2.5 mol% DMPE-PEG2000 was added to the liposomes to stabilize against aggregation and reduce the formation of multilamellar structures. The contribution of the PEGylated lipids to the overall scattering intensity is described by the analytical model presented by Arleth *et al*,[S5] and an implementation of this into the more detailed scattering density profile (SDP) model is also presented in detail by Nielsen *et al*[S6] for liposomes with incorporated peptide. Briefly, the scattering intensity for the liposomes which include a small fraction of PEGylated lipids the scattering intensity can be expressed as:

$$I_{lip-PEG} = I_{lip}(Q) + I_{chain}(Q) + I_{c_ic_i}(Q) + I_{c_oc_o}(Q) + I_{c_ic_o}(Q) + I_{sc_i}(Q) + I_{sc_o}(Q) \tag{S19}$$

where the first term on the right hand side, $I_{lip}(Q)$ is the liposome scattering as described above (Eq (S12)), the second term $I_{chain}(Q)$ is the scattering from the PEG-chains by themselves, modeled as random Gaussian coils. The next two terms, $I_{c_ic_i}(Q)$ and $I_{c_oc_o}(Q)$ are the interference terms between the individual chains on the inner and outer leaflet, respectively. $I_{c_ic_o}(Q)$ is then the inter-interference terms between chains on the inner and outer leaflets, while the last two terms, $I_{sc_i}(Q)$ and $I_{sc_o}(Q)$ are the interference terms between the inner and outer chains and the lipid bilayer, respectively. For the functional forms of these terms and a comprehensive discussion around them it is refereed to Refs.[S5,S6]

In this model the free parameters are the inner liposome radius, R_i, thickness t_j and the deviation in the DW-factor σ_j for each of the three shell, as well as the deviation σ_{RiPD} in the distribution function G. In addition the fraction of peptide in each shell, f_j^P is treated as a free parameter, but with the additional restriction that $\sum_j f_j^P = 1$. Although these parameters are essentially free, they are restricted to physically meaningful values in the fitting procedure. The remaining parameters are either calculated, such as the component scattering length densities and molar ratios, or taken from independent measurements or literature values, as is the case for the molecular volumes, and kept fixed in the fitting.

S2.1. Hydrocarbon shell polydispersity

In case of the raft-forming liposomes there is a substantial difference in the thickness of the bilayer in the raft phase and the continuous phase, as reported by Heberle *et al.*[S4] This is particularly evident in the decreased steepness of the SAXS scattering curve before the second minima, at intermediate to high Q. This thickness difference is likely increased further with the addition of peptide. To account for this, rather than using a single value for the hydrocarbon shell thickness, a Gaussian distribution of thicknesses is implemented, with average thickness $\overline{t_c}$ and deviation $\sigma_{cPD} \cdot \overline{t_c}$, the intensity accounting for this polydispersity is then given as:

$$I_{HC,PD} = \int_{t_{c,min}}^{t_{c,max}} \left[\frac{1}{\sigma_{cPD} \cdot \overline{t_c} \cdot \sqrt{2\pi}} \cdot \exp\left(\frac{-(t-\overline{t_c})^2}{2(\sigma_{cPD} \cdot \overline{t_c})^2} \right) \right] \cdot A_{CS}(Q,t)^2 \, dt \tag{S20}$$

where $A_{CS}(Q,t)$ is concentric shells amplitude (Eq. (S13)) evaluated at a given thickness value t, which ranges in the interval from $t_{c,min} = \overline{t_c} - 3 \cdot \sigma_{cPD} \cdot \overline{t_c}$ to $t_{c,max} = \overline{t_c} + 3 \cdot \sigma_{cPD} \cdot \overline{t_c}$. This integral over the hydrocarbon shell thickness is evaluated prior to that over the liposome radius presented in equation (S12).

The hydrocarbon shell polydispersity for the raft forming samples as a function of PL ratio is shown in figure S4.

S2.2. Multilamellarity

The multilamellarity observed for the liposomes comprised entirely of the unsaturated POPC or DOPC lipids (excluding a 2.5 molar % of PEG-ylated DMPE), is described by a paracrystalline structure factor[S7] expressed as:

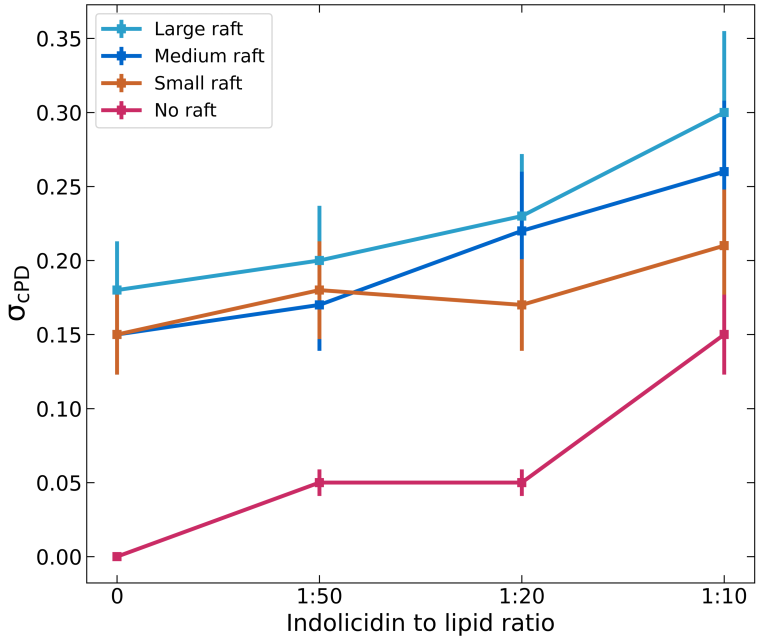

Figure S4: The standard deviation, σ_{cPD}, of the hydrocarbon shell thickness, resulting from fitting the SAXS model to the experimental data from the raft forming vesicles as well as the no raft control (Experimental SAXS data and model fits shown in figure 3).

$$S_{PC}(Q,n) = \left(n + 2\sum_{k=1}^{n-1}(n-k)\cos(kQd)\exp\left(-\frac{kQ^2\Delta^2}{2}\right)\right) + N_{diff}, \tag{S21}$$

where n is the number of bilayers, d is the separation distance between the bilayers and N_{diff} is the number of uncorrelated bilayers which contribute with an increased background. The paracrystalline theory accounts for small variations in the bilayer separation through the Debye-Waller temperature factor, Δ, as described in Ref.[S8] For bilayer stacks that are not completely well defined, two reasonable sources of polydispersity arise that reduce the number Bragg peaks and their sharpness. First, the average spacing between the layers can vary, and secondly the number of layers per multilamellar vesicle can vary. Both are taken into account by using a distribution around a mean value for the relevant variable.

If the bilayer stacking distance is distributed around an average value d and uncertainty σ_d, the structure factor can be expressed as:

$$S_{PC,PD}(Q,n) = \left(n + 2\sum_{k=1}^{n-1}\frac{\sum_{d_i=d-2\sigma_d}^{d+4\sigma_d}(n-k)\cos(kQd_i)\exp\left(-\frac{kQ^2\Delta^2}{2}\right)\cdot\exp\left[\frac{-(d-d_i)^2}{2\sigma_d^2}\right]}{\sum_{d_i=d-2\sigma_d}^{d+4\sigma_d}\exp\left[\frac{-(d-d_i)^2}{2\sigma_d^2}\right]}\right) + N_{diff}, \tag{S22}$$

where d_i is taken from the interval $[d-2\cdot\sigma_d, d+4\cdot\sigma_d]$.

To account for a distribution in number of stacked bilayers the structure factor, $S_{PC,PD}$ is convoluted with a normal distribution of the number of stacks around a mean N as:

$$S(Q) = \sum_{n=N-2\sigma}^{N+2\sigma} \left(\frac{1}{\sigma\sqrt{2\pi}} \cdot \exp\left[-\frac{(n-N)^2}{2\sigma^2} \right] \right) \cdot S_{PC,PD}(Q,n) \tag{S23}$$

while σ is the deviation of the normal distribution is defined as:

$$\sigma = \begin{cases} \sqrt{N} & \text{for } N \geq 5 \\ \frac{1}{2}(N-1) & \text{for } 2 \leq N < 5 \end{cases} \tag{S24}$$

σ this does not represent a new fitting parameter,[S7] as it is only defined in terms of the average number of stacks N.

The multilamellar structure factor is incorporated into the overall model by defining a fraction of unilamellar liposomes as f_{uni} and incorporating it into equation (S12) as:

$$I(Q) = n \int_0^\infty G(R_i, \sigma_{RiPD}) \cdot \left[A_{CS}(Q,R_i)^2 \left(f_{uni} + (1 - f_{uni}) S(Q) \right) \right] dR_i, \tag{S25}$$

The multilamellar features, although clearly present, are not very sharp in the single-lipid DOPC and POPC vesicles, and the experimental data was generally fitted using a low fraction of multilamellar liposomes and a small average number of stacks. Furthermore, as both the Debye-Waller temperature factor Δ and the polydispersity in the average staking distance σ_d smear out the Bragg peaks in similar ways, it was difficult to separate the two contributions and both could be used to explain the data. Hence, to reduce the number of free fitting parameters, Δ was kept at a fixed low value, while σ_d was treated as a fitting parameter.

S2.3. Liposome and micelle coexistence model

In case of the single-lipid liposome made up of saturated DSPC, significant solubilisation of the bilayer was observed at hight peptide:lipid ratios. Without a dedicated study it is not possible to determine the exact shape, structure and composition of the mixed micelles. Therefore, to limit the number of additional parameters and reduce the risk of overparameterisation, it is assumed that the micelles have the same molar ratio of lipids and peptide as for the liposomes in general, and are modeled as homogeneous spheres of radius R_M.

The scattering intensity from the micelle is then:

$$I_M(Q, R_M) = n_M \cdot \Delta\rho_M^2 \cdot V_M^2 \cdot A(Q, R_M)^2 \tag{S26}$$

where $A(Q, R_M)$ is the solid sphere amplitude (Eq. (S1)), $V_M = \frac{4\pi}{3} R_M^3$ is the volume of the micelle, and $\Delta\rho_M$ is the SLD contrast between the micelle and the solvent, determined from the average composition of the micelle through the volume fractions of lipids and peptide and their SLDs, similar to how it is done for the liposome itself. With the simplifying assumption that the molar ratios of the different components are the same for both the micelles and liposomes, no new parameters are introduced in the calculation of the micelle contrast. n_M is the number density of micelles in the system, and is calculated through the fraction of lipids (keeping the molar ratios constant, this also directly determines fraction of

peptides) that are in micelles, $f_{lip-in-M}$. Correspondingly, the number density of liposomes in the sample, n_{lip} is then determined from the fraction of lipids in liposomes, $1 - f_{lip-in-M}$

Finally, the total scattering intensity is given by:

$$I_{tot}(Q) = n_{lip} \cdot I_{lip-PEG} + n_M \cdot I_M \tag{S27}$$

S3. Simultaneous SAXS and SANS analysis

The simultaneous analysis of the SAXS and the two SANS curves with different contrast presented in figure 3 of the main manuscript, places much more stringent requirements for the fitted model parameters. In the analysis, all fit parameters (**shown in table S4**) are kept the same for the three contrast except for the scattering lengths of the lipids, peptide and buffer, which are explicitly calculated based on buffer and scattering condition and not fitted. As the peptide inserts into the bilayer, it partially partitions into the lipid tails, increasing average SLD of the tails and reducing the contrast to the heads, which consequently lowers the scattering intensity at low Q. Importantly, all three curves maintain their radial symmetry and can be well described over the whole extensive Q range. This strongly suggests that the peptides do no not cluster in the bilayer, but distribute rather uniformly in both phases.

S4. Single lipid vesicles

In addition to the raft forming vesicles and the separate L_o and L_d phases, (SAXS curves presented in figure 4 of the main manuscript), the structure of LUVs with single lipid compositions comprised of DSPC, POPC or DOPC, respectively were investigated. The SAXS results depicted in Fig. S5, show similar trends and the analysis show clear peptide insertion with tendency to accumulate on the outer leaflet for all three lipids. This again indicates that the peptide interacts with both phases, and even the completely saturated DSPC membrane. Although a small amount of free peptide may be present in solution, it is undetectable even at the double 1:10 PL ratio. The SAXS curves show that the vesicles remain intact and are not solubilized by the peptide, except for single lipid LUVs composed of saturated DSPC at 1:10 PL. (Fig S5A). The reduction of the maxima at intermediate Q, and the increasingly shallow minima with increasing peptide concentration, are characteristic of partial solubilization with co-existence of vesicles and much smaller mixed peptide:lipid micelles.[S9,S10] In the case of the raft forming vesicles, the bilayer thickness is between that of pure DSPC, DOPC, and POPC membranes, as expected

S5. SAXS model parameters and values

The tables below show the model parameters and values obtained from fitting the aforementioned models to the experimental SAXS data. Table S3 shows an overview over the relevant parameters, with the corresponding units and parameter explanations. It is emphasized that not all SAXS data was fitted with all the different additions to the model, such as multilamellarity or micelle coexistence. These features were only implemented when necessary and the simpler models were inadequate to explain all the features in the experimental curves. Hence, only the relevant and used parameters are displayed in the subsequent tables. Table

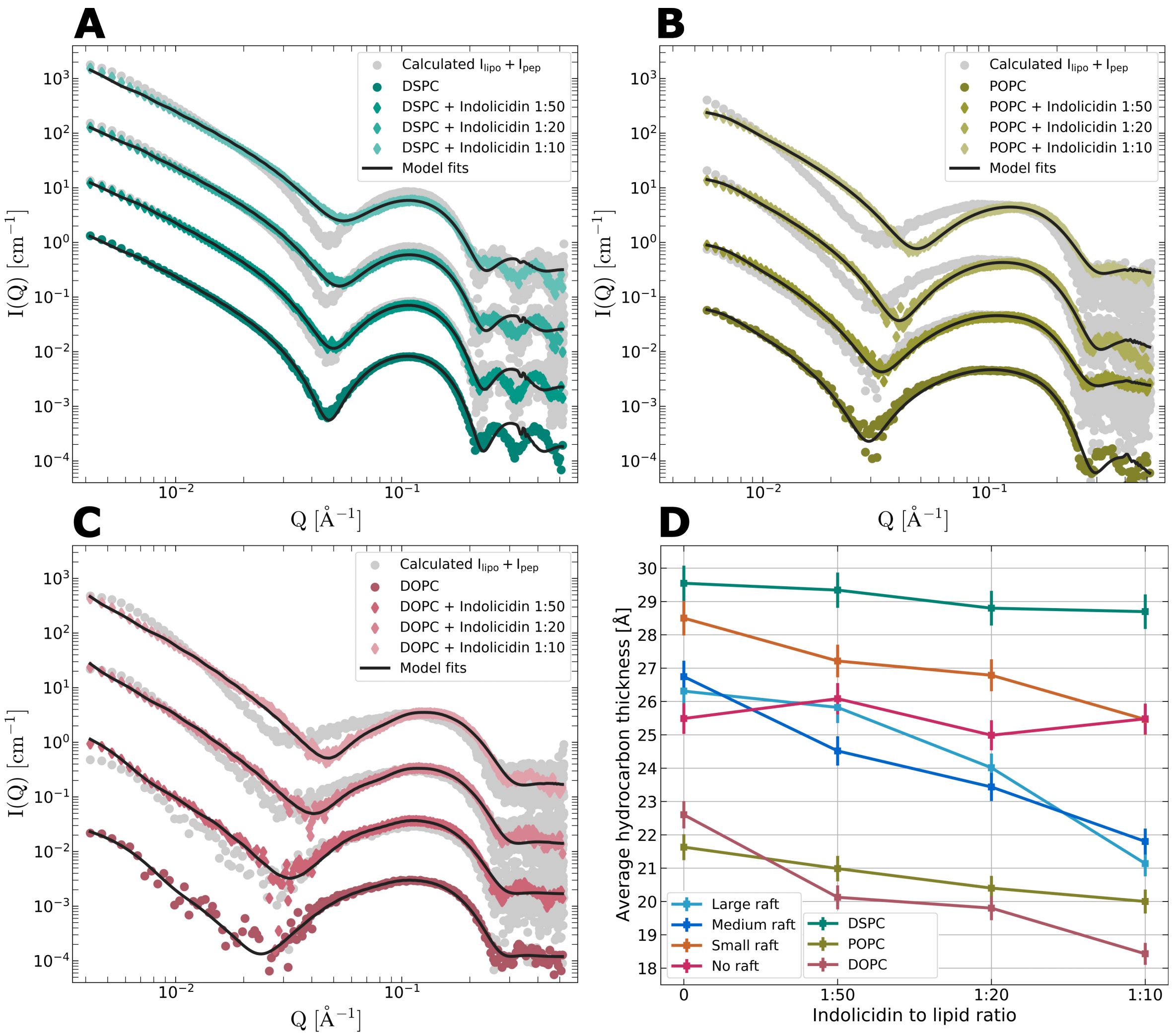

Figure S5: SAXS curve with model fits (solid lines) of single lipid vesicles comprised of DSPC (**A**), POPC (**B**) and DOPC (**C**) respectively. The average hydrocarbon thickness of the single lipid and raft forming vesicles are shown in panel**D**.

S5 shows the fit parameters for the vesicles forming large and medium rafts without added peptide to the left and increasing amounts of Indolicidin going to the right, the corresponding data for the vesicles forming small rafts and the non-raft forming control are shown in table S6. Table S8 shows the fit parameters for the single lipid DOPC and POPC vesicles with no peptide and with increasing amounts of added Indolicidin, while table S9 corresponds to the single lipid DSPC vesicles. Finally table S10 shows the fit parameters for the second batch of large raft forming vesicles without peptide and with the addition of LL-37, Magainin II and Aurein 2.2 in 1:100, 1:20 and 1:20 PL molar ratios, respectively.

The uncertainties presented in the tables below are estimated based on the maximum amount the given parameter can vary within a 10 % deviation of χ^2, when each of the remaining fitting parameters are individually relaxed.

Table S3: Overview over model parameters. **Parameter type** refers to how the parameter is treated in the fitting process with; F) Free fitting parameter, although constrained to not give nonphysical results. C) Calculated from other parameters . E) Given by the experimental parameters and allowed to deviate slightly. L) Based on literature values,[S11,S12] and allowed to slightly vary. N) A small and fixed value is chosen and not allowed to vary during the fitting procedure

**Parameter**	**Unit**	**Explanation**	**Parameter type**
R_i	[Å]	Inner vesicle radius	F
t_i	[Å]	Inner shell thickness	F
t_o	[Å]	Outer shell thickness	F
t_c	[Å]	Hydrocarbon (HC) shell thickness	F
σ_c	[Å]	DW smearing of HC shell thickness	F
σ_{t_i}	[Å]	DW smearing of inner shell thickness	F
σ_{t_o}	[Å]	DW smearing of outer shell thickness	F
ϕ_i^W	no units	Vol. fraction of water in inner shell	C
ϕ_o^W	no units	Vol. fraction of water in outer shell	F
c_L	[mg/mL]	Concentration of liposomes in sample	E
c_P	[mg/mL]	Concentration of peptide in sample	E
r_{PL}	no units	Molar ratio of peptide to lipids	C
$f_{P,free}$	no units	Fraction of free peptide	F
V_{lipid}	[Å^3]	Volume of average lipid molecule	L
V_{head}	[Å^3]	Volume of lipid head	L
f_o^P	no units	Fraction of peptide in outer shell	F
f_c^P	no units	Fraction of peptide in HC shell	F
f_o^P	no units	Fraction of peptide in inner shell	F
σ_{cPD}	no units	Standard div. of HC shell thickness	F
σ_{RiPD}	no units	Standard div. of inner vesicle radius	F
f_{uni}	no units	Fraction of unilamellar vesicles	F
Δ	[Å]	DW temperature factor in Paracrystalline MLV model	N
d	[Å]	Average separation between multilamellar bilayers	F
σ_d	[Å]	Uncertainty in average bilayer separation	F
N_{diff}	no unit	Number of uncorrelated bilayers	N
f_{LinM}	no unit	Fraction of lipids that are in micelles rather than vesicles	F
R_M	[Å]	Radius of spherical micelle	F

Table S4: Model parameters for the simultaneous fits of SANS and SAXS data (show in figure 3 of the main paper). Only the top 4 parameter; the lipid head and tail scattering lengths $\mathrm{SL_{Lhead}}$ and $\mathrm{SL_{Ltail}}$ respectively, and the SLDs of the buffer and peptide $\rho_{solvent}$ and $\rho_{peptide}$, respectively, differ for the three respective contrasts.

**Parameter**	**Large raft no pep**			**Large raft Indo 1:20**		
Contrast*	SANS 63.2%	SANS 21.3%	SAXS	SANS 63.2%	SANS 21.3%	SAXS
$\mathrm{SL_{Lhead}} \cdot 10^{-12}$ [cm]	6.01514	6.01514	0.45999	6.01514	6.01514	0.45999
$\mathrm{SL_{Ltail}} \cdot 10^{-12}$ [cm]	-3.01095	-3.01095	0.74866	-3.01095	-3.01095	0.74866
$\rho_{solvent} \cdot 10^{10}$ [cm^{-2}]	3.812	0.974	9.430	3.812	0.974	9.430
$\rho_{peptide} \cdot 10^{10}$ [cm^{-2}]	n.a	n.a	n.a	2.09	2.09	12.2
R_i [Å]		345 ± 22			260 ± 16.5	
t_i [Å]		6.0 ± 1.25			6 ± 1.25	
t_o [Å]		5.5 ± 0.69			6 ± 0.75	
t_c [Å]		27.0 ± 0.32			23.9 ± 0.3	
σ_c [Å]		4.5 ± 0.45			3 ± 0.38	
σ_{t_i} [Å]		8 ± 0.45			9 ± 0.70	
σ_{t_o} [Å]		8 ± 0.75			8 ± 0.75	
ϕ_i^W		0.275			0.344	
ϕ_o^W		0.290			0.282	
c_L [mg/mL]		10			10	
c_P [mg/mL]		n.a			1.29	
r_{PL}		n.a			0.050	
$f_{P,free}$		n.a			0	
V_{lipid} [Å^3]		1248 ± 1.3			1260 ± 1.5	
V_{head} [Å^3]		320 ± 3.5			330 ± 4.0	
f_o^P		n.a			0.7 ± 0.045	
f_c^P		n.a			0.3 ± 0.045	
f_i^P		n.a			0	
f_{uni}		0.85 ± 0.06			$0.99 \pm 0.05^*$	
N		3 ± 0.4			3 ± 0.4	
Δ		1			1	
d		130.0 ± 6.3			122 ± 8.4	
σ_d		24 ± 3.6			20 ± 4.9	
N_diff		0			0	
σ_{cPD}		0.18 ± 0.09			0.25 ± 0.013	
σ_{RiPD}		0.33 ± 0.023			0.52 ± 0.035	

Table S5: Model parameters for vesicles with large rafts (L) and medium rafts (M). For samples with added peptide the P:L molar ratios are indicated as 1:50, 1:20 and 1:10, respectively

**Parameter**	**L no pep**	**L 1:50**	**L 1:20**	**L 1:10**	**M no pep**	**M 1:50**	**M 1:20**	**M 1:10**
R_i [Å]	315 ± 38	300 ± 36	275 ± 33	320 ± 39	239 ± 29	400 ± 48	450 ± 54	450 ± 54
t_i [Å]	5.5 ± 1.1	5.5 ± 1.1	5.5 ± 1.1	5.5 ± 1.1	5.5 ± 1.1	5.5 ± 1.1	5.5 ± 1.1	5.5 ± 1.1
t_o [Å]	5.5 ± 0.35	6 ± 0.38	6 ± 0.38	6 ± 0.38	5.5 ± 0.35	5.5 ± 0.35	6 ± 0.38	6 ± 0.38
t_c [Å]	26.3 ± 0.48	25.8 ± 0.47	24 ± 0.43	21.1 ± 0.38	26.7 ± 0.48	24.5 ± 0.44	23.4 ± 0.42	21.8 ± 0.39
σ_c [Å]	3 ± 0.62	2 ± 0.41	1.84 ± 0.38	2 ± 0.41	2.88 ± 0.6	2.16 ± 0.45	1.28 ± 0.27	1.76 ± 0.37
σ_{t_i} [Å]	8 ± 0.65	8 ± 0.65	8 ± 0.65	7.5 ± 0.61	7 ± 0.57	9 ± 0.74	9 ± 0.74	8.8 ± 0.72
σ_{t_o} [Å]	10 ± 0.37	9 ± 0.33	10 ± 0.37	11 ± 0.41	11 ± 0.41	11 ± 0.41	11 ± 0.41	10.5 ± 0.39
ϕ_i^W	0.251	0.28	0.334	0.265	0.229	0.309	0.359	0.413
ϕ_o^W	0.332	0.352	0.318	0.272	0.336	0.3	0.312	0.198
c_L [mg/mL]	2.5	2.5	2.5	2.33	2.5	2.5	2.5	2.5
c_P [mg/mL]	n.a.	0.128	0.321	0.641	n.a.	0.129	0.322	0.643
r_{PL}	n.a.	0.02	0.05	0.107	n.a.	0.02	0.05	0.1
$f_{P,free}$	n.a.	0	0	0	n.a.	0	0	0
V_{lipid} [Å^3]	1225 ± 1.2	1230 ± 1.2	1233 ± 1.2	1238 ± 1.2	1217 ± 1.2	1218 ± 1.2	1222 ± 1.2	1233 ± 1.2
V_{head} [Å^3]	307.1 ± 4	305 ± 4	307 ± 4	308 ± 4	305 ± 4	310.4 ± 4	307.6 ± 4	307.6 ± 4
f_o^P	n.a.	0.7 ± 0.06	0.7 ± 0.06	0.6 ± 0.052	n.a.	0.7 ± 0.06	0.7 ± 0.06	0.8 ± 0.069
f_c^P	n.a.	0.3 ± 0.06	0.3 ± 0.06	0.1 ± 0.02	n.a.	0.3 ± 0.06	0.3 ± 0.06	0.2 ± 0.04
f_i^P	n.a.	0	0	0.3	n.a.	0	0	0
σ_{cPD}	0.18 ± 0.033	0.20 ± 0.037	0.23 ± 0.042	0.30 ± 0.055	0.15 ± 0.027	0.17 ± 0.031	0.22 ± 0.04	0.26 ± 0.048
σ_{RiPD}	0.39 ± 0.055	0.48 ± 0.068	0.50 ± 0.07	0.50 ± 0.07	0.48 ± 0.068	0.50 ± 0.07	0.5 ± 0.07	0.50 ± 0.07

Table S6: Model parameters for vesicles with small rafts (S) and the no raft control (NoR). For samples with added peptide the P:L molar ratios are indicated as 1:50, 1:20 and 1:10 respectively.

**Parameter**	**S no pep**	**S 1:50**	**S 1:20**	**S 1:10**	**NoR no pep**	**NoR 1:50**	**NoR 1:20**	**NoR 1:10**
R_i [Å]	192 ± 23	350 ± 42	450 ± 54	450 ± 54	380 ± 46	450 ± 54	450 ± 54	380 ± 46
t_i [Å]	5.5 ± 1.1	5.5 ± 1.1	5.5 ± 1.1	5.5 ± 1.1	5.5 ± 1.1	5.5 ± 1.1	5.5 ± 1.1	6.5 ± 1.3
t_o [Å]	5.5 ± 0.35	5.5 ± 0.35	6 ± 0.38	6 ± 0.38	6 ± 0.38	5.5 ± 0.35	5.5 ± 0.35	5.6 ± 0.36
t_c [Å]	28.5 ± 0.52	27.2 ± 0.49	26.8 ± 0.48	25.5 ± 0.46	25.5 ± 0.46	26.1 ± 0.47	25 ± 0.45	25.5 ± 0.46
σ_c [Å]	1.5 ± 0.31	1.5 ± 0.31	1.5 ± 0.31	1.5 ± 0.31	2.19 ± 0.45	2.5 ± 0.52	2.5 ± 0.52	1.2 ± 0.25
σ_{t_i} [Å]	5.5 ± 0.45	6 ± 0.49	6.3 ± 0.52	6.3 ± 0.52	10.3 ± 0.84	7.9 ± 0.65	8.5 ± 0.69	4.8 ± 0.39
σ_{t_o} [Å]	11 ± 0.41	11.4 ± 0.42	11 ± 0.41	11 ± 0.41	12.4 ± 0.46	12.4 ± 0.46	12.5 ± 0.46	12 ± 0.44
ϕ_i^W	0.148	0.234	0.264	0.334	0.392	0.394	0.428	0.512
ϕ_o^W	0.296	0.238	0.192	0.139	0.492	0.398	0.329	0.21
c_L [mg/mL]	2.5	2.5	2.5	2.5	2.5	2.5	2.5	2.5
c_P [mg/mL]	n.a.	0.129	0.322	0.644	n.a.	0.138	0.345	0.691
r_{PL}	n.a.	0.02	0.05	0.1	n.a.	0.02	0.05	0.1
$f_{P,free}$	n.a.	0	0	0	n.a.	0	0	0
V_{lipid} [Å^3]	1213 ± 1.2	1214 ± 1.2	1222 ± 1.2	1237 ± 1.2	1210 ± 1.2	1219 ± 1.2	1225 ± 1.2	1241 ± 1.2
V_{head} [Å^3]	307.4 ± 4	306 ± 4	307.4 ± 4	307.4 ± 4	300.9 ± 3.9	300 ± 3.9	300.4 ± 3.9	300 ± 3.9
f_o^P	n.a.	0.7 ± 0.06	0.8 ± 0.069	0.7 ± 0.06	n.a.	0.5 ± 0.043	0.7 ± 0.06	0.7 ± 0.06
f_c^P	n.a.	0.3 ± 0.06	0.2 ± 0.04	0.3 ± 0.06	n.a.	0.5 ± 0.1	0.3 ± 0.06	0.3 ± 0.06
f_i^P	n.a.	0	0	0	n.a.	0	0	0
σ_{cPD}	0.15 ± 0.027	0.18 ± 0.033	0.17 ± 0.031	0.21 ± 0.038	0	0.05 ± 0.009	0.05 ± 0.009	0.15 ± 0.027
σ_{RiPD}	0.50 ± 0.07	0.50 ± 0.07	0.48 ± 0.068	0.40 ± 0.056	0.50 ± 0.07	0.50 ± 0.07	0.50 ± 0.07	0.40 ± 0.056

Table S7: Model parameters for vesicles with composition corresponding the L_o and L_d phases of large raft forming vesicles. For samples with added Indolicidin the P:L molar ratios are indicated as 1:50, 1:20 and 1:10 respectively.

**Parameter**	L_o **no pep**	L_o **1:50**	L_o **1:20**	L_o **1:10**	L_d **no pep**	L_d **1:50**	L_d **1:20**	L_d **1:10**
R_i [Å]	239 ± 29	348 ± 42	348 ± 42	305 ± 37	271 ± 33	252 ± 30	260 ± 31	260 ± 31
t_i [Å]	5.5 ± 1.1	5.5 ± 1.1	5.5 ± 1.1	5.5 ± 1.1	6 ± 1.2	6 ± 1.2	5.5 ± 1.1	5.5 ± 1.1
t_o [Å]	5.5 ± 0.35	5.5 ± 0.35	5.5 ± 0.35	5.99 ± 0.38	5.5 ± 0.35	6 ± 0.38	6 ± 0.38	6 ± 0.38
t_c [Å]	27.7 ± 0.5	27.7 ± 0.5	27.5 ± 0.5	27.5 ± 0.5	23.1 ± 0.42	22.5 ± 0.41	21.2 ± 0.38	19.8 ± 0.36
σ_c [Å]	2.71 ± 0.56	2.71 ± 0.56	3.64 ± 0.76	3.51 ± 0.73	2.88 ± 0.6	2.84 ± 0.59	2.47 ± 0.51	2.65 ± 0.55
σ_{t_i} [Å]	9 ± 0.74	9 ± 0.74	9 ± 0.74	6.7 ± 0.55	5 ± 0.41	6.5 ± 0.53	7 ± 0.57	8 ± 0.65
σ_{t_o} [Å]	12.2 ± 0.45	12.2 ± 0.45	12.2 ± 0.45	12.3 ± 0.45	8.9 ± 0.33	9 ± 0.33	9.2 ± 0.34	9 ± 0.33
ϕ_i^W	0.251	0.275	0.29	0.318	0.369	0.384	0.383	0.395
ϕ_o^W	0.355	0.269	0.156	0.117	0.389	0.383	0.377	0.263
c_L [mg/mL]	2.5	2.5	2.5	2.5	2.5	2.5	2.5	2.5
c_P [mg/mL]	n.a.	0.131	0.328	0.655	n.a.	0.123	0.308	0.616
r_{PL}	n.a.	0.02	0.05	0.0999	n.a.	0.02	0.05	0.1
$f_{P,free}$	n.a.	0	0	0	n.a.	0	0	0
V_{lipid}	1220 ± 1.2	1220 ± 1.2	1228 ± 1.2	1251 ± 1.3	1252 ± 1.3	1252 ± 1.3	1260 ± 1.3	1262 ± 1.3
V_{head}	307.4 ± 4	307.4 ± 4	307.4 ± 4	307 ± 4	312.9 ± 4.1	312.9 ± 4.1	310.9 ± 4	310.9 ± 4
f_o^P	n.a.	0.8 ± 0.069	0.85 ± 0.073	0.782 ± 0.067	n.a.	0.935 ± 0.08	0.705 ± 0.061	0.8 ± 0.069
f_c^P	n.a.	0.2 ± 0.04	0.15 ± 0.03	0.218 ± 0.044	n.a.	0.0647 ± 0.013	0.245 ± 0.049	0.1 ± 0.02
f_o^P	n.a.	0	0	0	n.a.	0	0.05 ± 0.01	0.1 ± 0.02
f_{uni}	1	1	1	1	0.7 ± 0.08	0.7 ± 0.08	0.81 ± 0.09	0.9 ± 0.1
N	n.a.	n.a.	n.a.	n.a.	3 ± 0.3	3 ± 0.3	3 ± 0.3	3 ± 0.3
Δ	n.a.	n.a.	n.a.	n.a.	1	1	1	1
d	n.a.	n.a.	n.a.	n.a.	30 ± 2.3	30 ± 2.3	36.2 ± 2.8	36.2 ± 2.8
N_{diff}	n.a.	n.a.	n.a.	n.a.	0	0	0	0
σ_d	n.a.	n.a.	n.a.	n.a.	20 ± 3	20 ± 3	19.5 ± 2.9	19.5 ± 2.9
σ_{RiPD}	0.612 ± 0.086	0.363 ± 0.051	0.42 ± 0.059	0.424 ± 0.06	0.358 ± 0.051	0.339 ± 0.048	0.376 ± 0.053	0.403 ± 0.057

Table S8: Model parameters for DOPC and POPC vesicles. For samples with added peptide the P:L molar ratios are indicated as 1:50, 1:20 and 1:10 respectively. *multilamellarity is only defined for $N \geq 2$ hence uncertainty in this case only applies in positive direction.

Parameter	DOPC	DOPC1:50	DOPC1:20	DOPC1:10	POPC	POPC1:50	POPC1:20	POPC1:10
R_i [Å]	200 ± 24	220 ± 27	350 ± 42	369 ± 45	450 ± 54	450 ± 54	450 ± 54	450 ± 54
t_i [Å]	5.5 ± 1.1	5.5 ± 1.1	6.5 ± 1.3	6.5 ± 1.3	6 ± 1.2	6 ± 1.2	6 ± 1.2	6 ± 1.2
t_o [Å]	5.5 ± 0.35	5.5 ± 0.35	6.5 ± 0.41	6.5 ± 0.41	6 ± 0.38	7 ± 0.45	6.5 ± 0.41	6 ± 0.38
t_c [Å]	22.6 ± 0.41	20.1 ± 0.36	19.8 ± 0.36	18.4 ± 0.33	21.6 ± 0.39	21 ± 0.38	20.4 ± 0.37	20 ± 0.36
σ_c [Å]	4.9 ± 1	4 ± 0.83	3.24 ± 0.67	2.2 ± 0.46	1.5 ± 0.31	1.5 ± 0.31	1 ± 0.21	1 ± 0.21
σ_{t_i} [Å]	6.5 ± 0.53	7 ± 0.57	6 ± 0.49	7 ± 0.57	8 ± 0.65	8 ± 0.65	8 ± 0.65	7.8 ± 0.64
σ_{t_o} [Å]	6.5 ± 0.24	8.5 ± 0.31	8.5 ± 0.31	8.5 ± 0.31	9 ± 0.33	9 ± 0.33	9 ± 0.33	7.8 ± 0.29
ϕ_i^W	0.237	0.345	0.463	0.453	0.326	0.361	0.407	0.428
ϕ_o^W	0.347	0.359	0.367	0.368	0.373	0.448	0.397	0.192
c_L [mg/mL]	2.5	2.5	2.5	2.5	2.3	2.44	2.44	2.52
c_P [mg/mL]	n.a.	0.114	0.286	0.572	n.a.	0.105	0.27	0.56
r_{PL}	n.a.	0.02	0.05	0.1	n.a.	0.0193	0.0498	0.0997
$f_{P,free}$	n.a.	0	0	0	n.a.	0	0	0
V_{lipid}	1274 ± 1.3	1268 ± 1.3	1264 ± 1.3	1269 ± 1.3	1208 ± 1.2	1210 ± 1.2	1213 ± 1.2	1215 ± 1.2
V_{head}	324.8 ± 4.2	320 ± 4.2	324.4 ± 4.2	321 ± 4.2	320 ± 4.2	320 ± 4.2	320 ± 4.2	320 ± 4.2
f_o^P	n.a.	0.8 ± 0.069	0.8 ± 0.069	0.6 ± 0.052	n.a.	0.6 ± 0.052	0.5 ± 0.043	0.7 ± 0.06
f_c^P	n.a.	0.2 ± 0.04	0.2 ± 0.04	0.2 ± 0.04	n.a.	0.4 ± 0.08	0.5 ± 0.1	0.3 ± 0.06
f_i^P	n.a.	0	0	0.2	n.a.	0	0	0
f_{uni}	0.93 ± 0.1	0.87 ± 0.097	0.668 ± 0.075	0.704 ± 0.079	0.745 ± 0.083	0.801 ± 0.09	0.896 ± 0.1	0.951 ± 0.11
N	$2 \pm 0.3^*$	$2 \pm 0.3^*$	$2 \pm 0.3^*$	$2 \pm 0.3^*$	3 ± 0.45	3 ± 0.45	3 ± 0.45	3 ± 0.45
Δ	1	1	1	1	1	1	1	1
d	120 ± 9.2	109 ± 8.4	110 ± 8.5	105 ± 8.1	23.4 ± 1.8	27 ± 2.1	27.5 ± 2.1	27 ± 2.1
σ_d	20 ± 3	18.9 ± 2.8	21.9 ± 3.3	21 ± 3.1	10 ± 1.5	10 ± 1.5	10 ± 1.5	10 ± 1.5
N_{diff}	0	0	0	0	0	0	0	0
f_{LinM}	0	0	0	0	0	0	0	0
R_M	n.a.	n.a.	n.a.	n.a.	n.a.	n.a.	n.a.	n.a.
σ_{cPD}	0	0	0	0	0	0	0	0
σ_{RiPD}	0.40 ± 0.056	0.50 ± 0.07	0.52 ± 0.073	0.52 ± 0.073	0.40 ± 0.056	0.40 ± 0.056	0.40 ± 0.056	0.40 ± 0.056

Table S9: Model parameters for DSPC vesicles. For samples with added peptide the P:L molar ratios are indicated as 1:50, 1:20 and 1:10 respectively.

Parameter	DSPC no pep	DSPC 1:50	DSPC 1:20	DSPC 1:10
R_i [Å]	550 ± 67	550 ± 67	550 ± 67	500 ± 60
t_i [Å]	5.5 ± 1.1	6 ± 1.2	6 ± 1.2	6 ± 1.2
t_o [Å]	6 ± 0.38	6 ± 0.38	6.5 ± 0.41	6.5 ± 0.41
t_c [Å]	29.5 ± 0.53	29.3 ± 0.53	28.8 ± 0.52	28.7 ± 0.52
σ_c [Å]	1 ± 0.21	1 ± 0.21	1 ± 0.21	1 ± 0.21
σ_{t_i} [Å]	5 ± 0.41	5 ± 0.41	5 ± 0.41	5 ± 0.41
σ_{t_o} [Å]	11.5 ± 0.42	11 ± 0.41	11 ± 0.41	11 ± 0.41
ϕ_i^W	0.0662	0.157	0.205	0.248
ϕ_o^W	0.203	0.115	0.152	0.0601
c_L [mg/mL]	2.5	2.5	2.5	2.5
c_P [mg/mL]	n.a.	0.114	0.285	0.569
r_{PL}	n.a.	0.02	0.05	0.1
$f_{P,free}$	n.a.	0	0	0
V_{lipid}	1275 ± 1.3	1274 ± 1.3	1279 ± 1.3	1291 ± 1.3
V_{head}	320 ± 4.2	319.3 ± 4.2	319.3 ± 4.2	319.3 ± 4.2
f_o^P	n.a.	0.876 ± 0.075	0.66 ± 0.057	0.635 ± 0.055
f_c^P	n.a.	0.124 ± 0.025	0.34 ± 0.068	0.365 ± 0.073
f_i^P	n.a.	0	0	0
f_{LinM}	0	0.18 ± 0.01	0.27 ± 0.015	0.34 ± 0.019
R_M	20 ± 1.9	18 ± 1.7	18 ± 1.7	18 ± 1.7
σ_{cPD}	0	0	0	0
σ_{RiPD}	0.40 ± 0.056	0.40 ± 0.056	0.40 ± 0.056	0.47 ± 0.066

Table S10: Model parameters for vesicles forming large rafts with different peptides added. LL37 is added in 1:100 PL ratio, while Magainin II (MagII) and Aurein 2.2 (Aur2.2) are both added in 1:20 PL ratios

**Parameter**	**L no pep**	**L LL37 1:100**	**L MagII 1:20**	**L Aur2.2 1:20**
R_i [Å]	345 ± 42	418 ± 51	429 ± 52	432 ± 52
t_i [Å]	5.5 ± 1.1	5.5 ± 1.1	5.5 ± 1.1	5.5 ± 1.1
t_o [Å]	5.5 ± 0.35	5.5 ± 0.35	6 ± 0.38	5.5 ± 0.35
t_c [Å]	26.3 ± 0.48	25.2 ± 0.46	25.6 ± 0.46	23.1 ± 0.42
σ_c [Å]	3 ± 0.62	2 ± 0.41	2.75 ± 0.57	1 ± 0.21
σ_{t_i} [Å]	6.08 ± 0.5	5.8 ± 0.47	6.24 ± 0.51	10 ± 0.82
σ_{t_o} [Å]	10.5 ± 0.39	11.5 ± 0.42	10.1 ± 0.37	10 ± 0.37
ϕ_i^W	0.26	0.286	0.26	0.358
ϕ_o^W	0.333	0.336	0.178	0.438
c_L [mg/mL]	2.5	2.5	2.5	2.5
c_P [mg/mL]	n.a.	0.0155	0.41	0.24
r_{PL}	n.a.	0.00103	0.0494	0.0494
$f_{P,free}$	n.a.	0	0	0
V_{lipid}	1227 ± 1.2	1214 ± 1.2	1241 ± 1.2	1234 ± 1.2
V_{head}	307 ± 4	306.5 ± 4	309.2 ± 4	307.7 ± 4
f_o^P	n.a.	0.70 ± 0.06	0.80 ± 0.069	0
f_c^P	n.a.	0.30 ± 0.06	0.10 ± 0.02	0.80 ± 0.16
f_i^P	n.a.	0	0.10 ± 0.02	0.20 ± 0.16
f_{uni}	1	1	1	1
Δ	n.a.	n.a.	n.a.	n.a.
d	n.a.	n.a.	n.a.	n.a.
σ_d	n.a.	n.a.	n.a.	n.a.
N_{diff}	n.a.	n.a.	n.a.	n.a.
f_{LinM}	0	0	0	0
R_M	n.a.	n.a.	n.a.	n.a.
σ_{cPD}	0.158 ± 0.029	0.21 ± 0.038	0.208 ± 0.038	0.22 ± 0.04
σ_{RiPD}	0.33 ± 0.047	0.34 ± 0.048	0.346 ± 0.049	0.37 ± 0.052

References

[S1] Oliveira, C. L. P.; Juul, S.; Jørgensen, H. L.; Knudsen, B.; Tordrup, D.; Oteri, F.; Falconi, M.; Koch, J.; Desideri, A.; Pedersen, J. S.; Andersen, F. F.; Knudsen, B. R. Structure of Nanoscale Truncated Octahedral DNA Cages: Variation of Single-Stranded Linker Regions and Influence on Assembly Yields. *ACS Nano* **2010**, *4*, 1367–1376.

[S2] Pedersen, J. Resolution effects and analysis of small-angle neutron scattering data. 1993; 9th International Conference on Small Angle Scattering ; Conference date: 27-04-1993 Through 30-04-1993.

[S3] Pedersen, J. In *Neutron, X-rays and light. Scattering methods applied to soft condensed matter*; Zemb, T., Lindner, P., Eds.; North-Holland: Oxford, England, 2002; Chapter 6, pp 127–144.

[S4] Heberle, F. A.; Petruzielo, R. S.; Pan, J.; Drazba, P.; Kučerka, N.; Standaert, R. F.; Feigenson, G. W.; Katsaras, J. Bilayer Thickness Mismatch Controls Domain Size in Model Membranes. *Journal of the American Chemical Society* **2013**, *135*, 6853–6859.

[S5] Arleth, L.; Vermehren, C. An analytical model for the small-angle scattering of polyethylene glycol-modified liposomes. *Journal of Applied Crystallography* **2010**, *43*, 1084–1091.

[S6] Nielsen, J. E.; Bjørnestad, V. A.; Lund, R. Resolving the structural interactions between antimicrobial peptides and lipid membranes using small-angle scattering methods: the case of indolicidin. *Soft Matter* **2018**, *14*, 8750–8763.

[S7] Kohlbrecher, J. *User guide for the SASfit software package*; Paul Scherrer Institute, June 21 2023; pp 660–663.

[S8] Pabst, G.; Koschuch, R.; Pozo-Navas, B.; Rappolt, M.; Lohner, K.; Laggner, P. Structural analysis of weakly ordered membrane stacks. *Journal of Applied Crystallography* **2003**, *36*, 1378–1388.

[S9] Bjørnestad, V. A.; Orwick-Rydmark, M.; Lund, R. Understanding the Structural Pathways for Lipid Nanodisc Formation: How Styrene Maleic Acid Copolymers Induce Membrane Fracture and Disc Formation. *Langmuir* **2021**, *37*, 6178–6188.

[S10] Nielsen, J. E.; Prévost, S. F.; Jenssen, H.; Lund, R. Impact of antimicrobial peptides on E. coli-mimicking lipid model membranes: correlating structural and dynamic effects using scattering methods. *Faraday discussions* **2021**, *232*, 203–217.

[S11] Greenwood, A. I.; Tristram-Nagle, S.; Nagle, J. F. Partial molecular volumes of lipids and cholesterol. *Chemistry and Physics of Lipids* **2006**, *143*, 1–10.

[S12] Pan, J.; Heberle, F. A.; Tristram-Nagle, S.; Szymanski, M.; Koepfinger, M.; Katsaras, J.; Kučerka, N. Molecular structures of fluid phase phosphatidylglycerol bilayers as determined by small angle neutron and X-ray scattering. *Biochimica et Biophysica Acta (BBA) - Biomembranes* **2012**, *1818*, 2135–2148.
